# Modeling and analysis of Hepatitis B dynamics with vaccination and treatment with novel fractional derivative

**DOI:** 10.1371/journal.pone.0307388

**Published:** 2024-07-18

**Authors:** Anum Zehra, Saba Jamil, Muhammad Farman, Kottakkaran Sooppy Nisar

**Affiliations:** 1 Department of Mathematics, The women university Multan, Multan, Pakistan; 2 Institute of Mathematics, Khwaja Fareed University of Engineering and Information Technology, Rahim Yar Khan, Pakistan; 3 Department of Mathematics, Faculty of Arts and Sciences, Near East University, Nicosia, Northern Cyprus, Turkey; 4 Department of Computer Science and Mathematics, Lebanese American University, Beirut, Lebanon; 5 Department of Mathematics, College of Science and Humanities, Prince Sattam bin Abdulaziz University, Alkharj, Saudi Arabia; Osaka University, JAPAN

## Abstract

These days, fractional calculus is essential for studying the dynamic transmission of illnesses, developing control systems, and solving several other real-world issues. In this study, we develop a Hepatitis B (HBV) model to observe the dynamics of vaccination and treatment effects to control the disease by using novel fractional operator. Modified Atangana-Baleanu-Caputo (MABC) is a new definition of the used derivative that is based on a modification of the Atangana and Baleanu derivatives. By employing the MABC fractional derivative, which incorporates the concepts of non-locality and memory effects our model captures the complex dynamics of HBV transmission more accurately than traditional models. An objective of this study is to analyze the effect of immunization and treatment techniques on the course of the hepatitis B virus, with a particular focus on the changing order of differentiation. Thereby, our paper deals with the stability analysis, positiveness, existence and uniqueness of the solution and simulations. Analysis of reproductive number *R*_0_ with the impact of different parameters is also treated. The proposed model’s existence and uniqueness findings are examined through the use of Banach’s fixed point and Leray-Schauder nonlinear alternative theorems. The equilibria for the models are determined to be globally stable using Lyapunov functions. The simulations for certain parameters are achieved by applying the Lagrange interpolation for the numerical computations and also the results are compared with the ABC operator results. The model is validated using numerical simulations, which are also used to assess how well different intervention techniques work to lower the impact of HBV infection and prevent its spread throughout the community. The results of this research assist in developing public health policies intended to lower the incidence of HBV infection worldwide and offer insightful information about how well treatment and vaccination strategies work to prevent HBV disease.

## 1 Introduction

Mathematical models can be used to illustrate the transmission of infectious diseases and the probable outcomes of a pandemic, which is particularly useful when making public health decisions. Models which consist of assumptions and pooled data which can be used to approximate real world data (as explained in papers [1–3]) use mathematics to develop criteria for various infectious diseases and output the impact of several interventions, including mass vaccination programs, asymptomatic and so on. Modeling will help us to establish which interventions will most likely be successful in preventing mortality and the endemic spread, as well as make clear which measures are appropriate and highlight example of measure implementation against others and the future tendencies, and so on [4]. Theoretical epidemiologists have been employing mathematical models to investigate the dynamics of disease transmission since Kermack and McKendrick [5]. Infection with the hepatitis B virus (HBV) is one of the world’s most deadly infections. It can lead to chronic liver disease and infection, as well as a high chance of dying from liver cancer. Hepatitis B infections only occur when the virus enters the bloodstream and travels to the liver. Rapid viral dissemination in the liver results in high production of new viruses that are released into the circulation. This infection primarily manifests in two stages: acute and chronic. The duration of an acute hepatitis B infection is less than six months. Should the infection be acute, your body’s immune system ought to be capable of eliminating the virus in a few months, and you should experience full recovery. The World Health Organization (WHO) estimates that 2 million people globally have hepatitis B virus (HBV) infection, and 350 million of those people are chronic carriers [6].

In this approach, mathematical models can be a useful tool that helps us maximize the use of limited resources or just make infection control techniques more effective. Mathematical modeling is crucial to studying many diseases in depth [7, 8]. Using mathematical modeling, several infectious diseases, including COVID-19 in its SIR form, were investigated in [9] to determine how quickly they spread and what precautions might be taken. A plan to eradicate HBV was developed in New Zealand using a mathematical model for Hepatitis B [10]. A mathematical model was developed by Anderson and May [11] to illustrate how carriers impact the transmission of HBV. An age structure model for forecasting HBV transmission dynamics was proposed by Zhao et al. [12], who also examined the immunization program’s long-term efficacy in China. Wang et al. [13] developed a finite domain diffusion model to study hepatitis B viral infection. An epidemic model that took Jaouade, immunization, and HBV infection transmission into account was developed by Khan and Zaman [14]. Farman et al. [15] presented an unconditionally convergent semi-analytical strategy based on contemporary evolutionary computational techniques and Pade approximation (EPA) for the treatment of nonlinear Hepatitis-B models.

Emergent fractional derivatives are defined in many ways in the literature. The Riemann-Liouville nonlocal structural derivative, which is derived from the Riemann-Liouville fractional derivative, retains the convolution operator for memory characterization while expanding the prediction and simulation range [16]. This is accomplished through its kernel function or structure-function. The Caputo fractional derivative [17] is one of Michele Caputo’s 1967 definitions that is most useful. The presence of a unique kernel is one of the primary issues with this variant. Caputo and Fabrizio [18] used a non-singular fractional derivative that depends on the exponential function to solve this problem. The result of replacing the single power-law kernel with a non-singular exponential kernel is this derivative, which is not sufficient to describe the non-exponential phenomenon. This is the reason why Atangana and Baleanu used the expanded Mittag-Leffler function [19] to formulate derivatives involving non-local and non-singular kernels. The Atangana-Baleanu-Caputo derivative (MABC) was proposed by Refai and Baleanu and also improved the Atangana and Baleanu fractional operator in the Caputo sense [20]. This operator can take help of its amazing features to solve the issues which can’t be solved by the Atangana and Baleanu operators.

The innovative ABC algorithm derivative is a great memory since it has convenient Mittag-Leffler function as its nonlocal kernel which makes its comparative performance better than other fractional derivative operators that are in the use now. A fractional derivative of the Caputo type is employed in the transmission of AIDS and HIV [21]. Rehman et al. [22] examined a fractional order model for COVID-19 and assessed the model’s behavior using three different derivatives: Caputo, Caputo-Fabrizio, and Atangana-Baleanu. In terms of stability, they discovered that Caputo outperformed the other two operators. In [23], utilizes the Mittag-Leffler stability idea and presents fractional Lyapunov functions for epidemic models. The global stability of the epidemic model in an equilibrium state is established. Inverse Mittag-Leffler functional based structural derivative was used [24] to explain the non-Gaussianity of ultraslow diffusion. For the long time limits, the nonlocal structural derivative diffusion was studied using inverse Mittag-Leffler function [25] which is an expression of the ultraslow diffusion in a dense colloid. The ABC operator (instead of others) approach would be applied and several scientific models including the General Sequential Hybrid Class of FDEs [26], the revolutionary finite difference [27], the modified fractional difference operators [28], the HIV/AIDS [29] and the Bovine Brucellosis disease model [30].

By utilizing fractional calculus, the model can add memory effects. Addressing the complexity of HBV dynamics, it should be noted that a person at different phases of the disease may have long lasting periods of different infection phases, and the previous infection of such individual could contribute immensely to the present stage of the disease. It seems that the ABC fractional derivative being capable of characterizing the memory phenomenon in a better way than integer-order derivatives, is a proper tool to explore the long-term dynamics in the course of HBV infection. This article explores an exceptional Hepatitis B model transmission in which the new Modified Atangana-Baleanu (MABC) operator is used. MABC as the altered form of the Atangana-Baleanu fractional derivative (ABC) instead of the usual approach has several unique aspects. The advantages of MABC method include the capturing of crossover behavior, the monitoring issue of modeling and the improvement in accuracy but it has limitations of parameter sensitivity and finding effective control strategy. Of all the methods, there is the possibility of this technique being both precisely simulating the HBV transmission dynamics and well evaluating the performance of interventions. The innovation of vaccination and treatment strategies in MABC model derivation creates novel avenues of assessment for epidemic models and establishment of the efficiency of interventions in the process of tackling diseases such as HBV. It is the first work among using the MABC operator to HBV dynamics and that of vaccination and treatment methods in order to get more inspirations for future research and exploring in this field. The MABC fractional derivative offers several advantages over existing methods:

- The MABC fractional derivative represents these effects more accurately and also captures the memory and nonlinear phenomena in disease spread, thus giving one a better understanding of disease dynamics.
- With MABC fractional derivatives we have new parameters that increase the system flexibility and accuracy for the HBV simulation. The flexibility of the strategy helps us to enforce effective control strategies aimed at dealing with the increasing number of cases in endemic model, which is comprised of different compartments.
- One of the main features of the MABC approach is that it allows to model the crossover behavior by considering the memory effects and non-local interactions which are characteristic in spread of such infectious diseases as the HBV ones. The MABC model being able to elaborate on these dynamics provide in depth knowledge on HBV transmission and in turn improve control strategy development.

The following is a breakdown of the paper’s structure. The introduction and historical context are covered in section one. The suggested techniques are defined in some basic terms in section two. The third section examines the fractional order model’s positivity, disease-free equilibrium, and endemic equilibrium. The fourth section uses the Lyapunov function to study the asymptotic stability of the fractional order HBV model globally. The existence and uniqueness of a system of solutions are verified in section five through the application of fixed point theory. The modified Atangana Baleanu in the Caputo sense fractional-order system is solved in section six using an innovative numerical approach. The numerical results of the proposed model are shown as graphs in section seven. The conclusion is included in section eight, which is the last section.

## 2 Basic concepts

We will review some fundamental fractional calculus concepts in this section.

**Definition 2.1** [20]

It is assumed that *g* ∈ *H*^1^([0, *T*]), *T* > 0, and *ν* ∈ (0, 1). For a function *g*(*t*), the ABC fractional derivative is presented as

0ABCDtνg(t)=B(ν)1-ν∫0tddσg(σ)Eν(-ν(t-σ)1-ν)dσ, (1)

**Definition 2.2** [20]

The definition of an ABC fractional integral for a function *g*(*t*) is

0ABCItνg(t)=1-νB(ν)g(t)+νB(ν)Γ(ν)∫0tg(σ)(t-σ)ν-1dσ, (2)

where *ν* ∈ (0, 1) and

$$\begin{array}{c} B\left(\nu \right)=1-\nu +\frac{\nu }{\Gamma (\nu )}. \end{array}$$ (3)

**Definition 2.3** [20]

For *g* ∈ *L*^1^(0, *T*) and *ν* ∈ (0, 1) the modified ABC derivative is defined by

MABCD0νg(t)=B(ν)1-ν[g(t)-Eν(-oνtν)g(0)-oν∫0t(t-σ)ν-1Eν,ν(-oν(t-σ)ν)g(σ)dσ], (4)

where $o_{\nu }=\frac{\nu }{1-\nu }$ and $B\left(\nu \right)=1-\nu +\frac{\nu }{\Gamma (\nu )}$.

The derivative of MABC has the following Laplace transform:

L{MABCDtνg(t);s}=B(ν)(1-ν)sνL{x(t);s}-sν-1g(0)sν+oν,|oνsν|<1. (5)

**Definition 2.4** [20]

For *g* ∈ *L*^1^(0, *T*) and *ν* ∈ (0, 1) the modified ABC integral is given as follow

MABCI0νg(t)=AB(1-ν)B(ν)[g(t)-g(0)]+oν[RLI0ν(g(t)-g(0))]. (6)

**Lemma 2.1** [20]

For *g*′(*t*) ∈ (0, ∞) and *ν* ∈ (0, 1) we have

MABCI0νMABCD0νg(t)=g(t)-g(0). (7)

## 3 Fractional order Hepatitis B model

In this section, we analyze the dynamic transmission of HBV models that is introduced by [31]. Fractional order derivative modified ABC is implemented to study the SEACTR model because its power to describe nonlinearity, anomaly, and memory effects of HBV transmission behaviors precisely. The MABC technique has advantages in modeling HBV transmission behavior as it allows for crossover behavior, and is more convenient in modeling, accurate in estimations, demonstrates sensitivity to changes in the parameters, and can lead to effective control strategies. These motivations focus the research on generating models that offer better comprehension and control of infectious diseases. The entire population is divided into six time-dependent compartments in this model based on the kinds of HBV infection, specifically S susceptible, E exposed, A acutely infected, C chronically infected, T treated and R recovered classes. Susceptible population grows with the arrival of the birth flux rate $\mu -\mu p_{1}C-\mu \theta R$, the rate of loss of immunity *u*_2_ from a resurgent population, and decrease with the infection force invading the exposed population. Proportions of susceptible moves into the recovered class by vaccination by the rate *τ* increases and. The number of people who are exposed grows as the infection spreads and reduces by *c*_1_ rate of transmission from exposed to acutely infected class and *c*_2_ rate of exposure to chronic infectious disease. The rate of transfer from being exposed to an acute infectious disease *c*_1_ raises the acute infective class, while the rate of transferring from acute to chronic class *δ*_1_ and the rate of natural death *μ* reduce it. From E to C transfer rate, as well as A to C class transition rate, raises the chronic infective class; we assume a proportional distribution *p*_1_ for vertical transmission of infants from infected class are infected, represented by the term $\mu p_{1}C,\left(p_{1}<1\right)$, and reduces by treatment rate *σ* and disease-induced death rate *d*. Treatment class rises with treatment rate *σ* and reduces with recovery rate *ϕ*. *θ*(< 1) represents the proportion *θ* of infants from the recovered class who are immune. The rates of recovery *ϕ*, vaccination rate *θ* with a proportion of *μ*, and proportions of susceptible individuals migrate to the recovered class by vaccination rate *τ*. Since vaccination is not perfect, this decreases by the loss of immunity rate *u*_2_.

- *c*_1_ is the rate at which those who are exposed become chronic HBV carriers.
- The rate at which exposed people spread to acutely infected persons is denoted by *c*_2_.
- The rate of transition from the acute infectious class to the chronic HBV carrier class is represented by *δ*_1_, and the rate of death from HBV illness is represented by *d*.
- The rate of chronically infected persons seeking treatment is denoted by *σ*.
- The rate at which acutely infectious persons recover naturally is denoted by *δ*_2_.
- The mass action term cω(A+γC)S represents the horizontal transmission of disease propagation. Here, *c* stands for per capita contact rate, *ω* represents the likelihood of contracting HBV infection per encounter with an infectious individual, and *γ* denotes the population’s level of chronic infectiousness.
- Let us assume that there is a proportion *p*_1_ of infected infants in the infected class in the event of vertical transmission. This proportion is expressed by the phrase $\mu p_{1}C,$ (*p*_1_ < 1)
- By natural death rate *μ*, each compartment declines.

**Fig 1:**
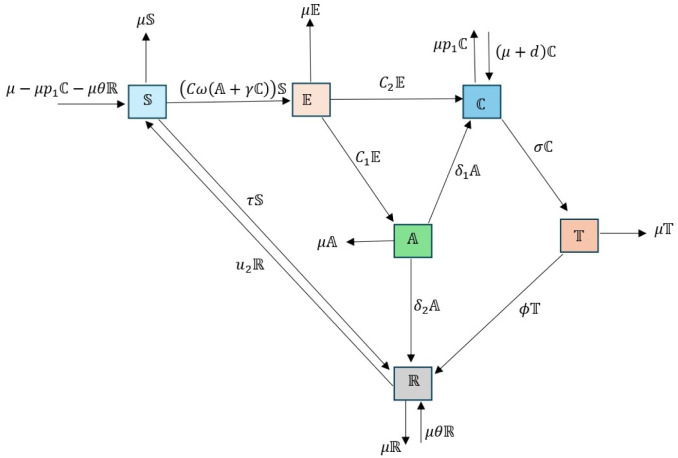
Schematic diagram of HBV model.

We make the assumption that there are fewer newborn carriers born to carriers than there are carriers who die and those who transition from a carrier state to a recovery state combined. Since carriers will continue to grow quickly as long as there is an infection, we get *μp*_1_ < *μ* + *d* + *σ* in this instance; that is, MABCDtνC>0 for C≠0 or A≠0. We constructed the corresponding flow chart depicted in Fig 1 based on the aforementioned assumptions. We constructed the corresponding dynamical system below based on the flow diagram shown in Fig 1.

{MABCDtνS=μ-μp1C-μθR+u2R-(cω(A+γC)+m1)S,MABCDtνE=cω(A+γC)S-m2E,MABCDtνA=c1E-m3A,MABCDtνC=c2E+δ1A-(m4-μp1)C,MABCDtνT=σC-m5T,MABCDtνR=ϕT+δ2A+τS+(μθ-m6)R, (8)

with initial conditions

$$\begin{array}{c} S(0)\geq 0,\;E(0)\geq 0,\;A(0)\geq 0,\;C(0)\geq 0,\;T(0)\geq 0,\;R(0)\geq 0, \end{array}$$ (9)

where

$$\begin{array}{c} m_{1}=\left(\mu +\tau \right),\;\;m_{2}=\left(c_{1}+c_{2}+\mu \right),\;\;m_{3}=\left(\delta_{1}+\delta_{2}+\mu \right),\;\;m_{4}=\left(\sigma +d+\mu \right),\\ \;m_{5}=\left(\phi +\mu \right),\;\;m_{6}=\left(\mu +u_{2}\right). \end{array}$$

### 3.1 Positivity analysis and equilibria

The determination of the disease-free equilibrium point and the analysis of the system’s stability at this point are among the most crucial issues to be solved in mathematical epidemiology models (8) to zero. When E=A=C=0, the disease-free steady state points can be found by solving the system

MABCDtνS=0,MABCDtνE=0,MABCDtνA=0,MABCDtνC=0,MABCDtνT=0,MABCDtνR=0.

Therefore, the disease-free steady state points are given by

$$\begin{array}{c} E^{0}=\left(\frac{\mu +u_{2}-\mu \theta }{\mu +\tau +u_{2}-\mu \theta },0,0,0,0,\frac{\tau }{\mu +\tau +u_{2}-\mu \theta }\right), \end{array}$$ (10)

and the endemic equilibrium points are $E_{1}=\left(S^{*},E^{*},A^{*},C^{*},T^{*}\right)$

$$\begin{array}{c} \begin{array}{c} S^{*}=\frac{\left(\mu +c_{1}\right)\left(\mu +c_{2}\right)\left(\mu +\delta_{2}-p_{1}\mu +u_{2}\right)}{c\omega c_{1}\left(\mu +\delta_{2}+\delta_{1}-p_{1}\mu +u_{2}\right)},\;E^{*}=\frac{c\omega \left(A^{*}+\gamma C^{*}\right)S^{*}}{\mu +c_{1}+c_{2}},\;A^{*}=\frac{c\omega c_{1}\gamma C^{*}S^{*}}{1-c\omega c_{1}S^{*}},\\ C^{*}=c_{2}c\omega (\frac{\left(A^{*}+\gamma C^{*}\right)S^{*}}{\left(\mu +c_{1}+c_{2}\right)\left(\mu +d+\sigma -\mu p_{1}\right)})+\frac{c\delta_{1}\omega c_{1}\theta C^{*}S^{*}}{\left(1-c\omega c_{1}S^{*}\right)\left(\mu +\sigma -\mu p_{1}\right)},\\ T^{*}=\frac{\tau }{\left(\varphi +\mu \right)}(\frac{c_{2}(c\omega \left(A^{*}+\gamma C^{*}\right)S^{*}}{\left(\mu +c_{1}+c_{2}\right)\left(\mu +\sigma -\mu p_{1}\right)})+\frac{c\delta_{1}\omega c_{1}\gamma C^{*}S^{*}}{\left(1-c\omega c_{1}S^{*}\right)\left(\mu +\sigma -\mu p_{1}\right)}. \end{array} \end{array}$$

Also from [31] we have reproductive number *R*_0_:

$$\begin{array}{c} R_{0}=\frac{c\omega \left(m_{6}-\mu \theta \right)\left(c_{1}m_{2}+\delta_{1}\gamma c_{1}+\gamma c_{2}\left(m_{4}-\mu p_{1}\right)\right)}{m_{2}m_{3}\left(m_{1}+u_{2}-\mu \theta \right)\left(m_{6}-\mu p_{1}\right)}. \end{array}$$

**Fig 2:**
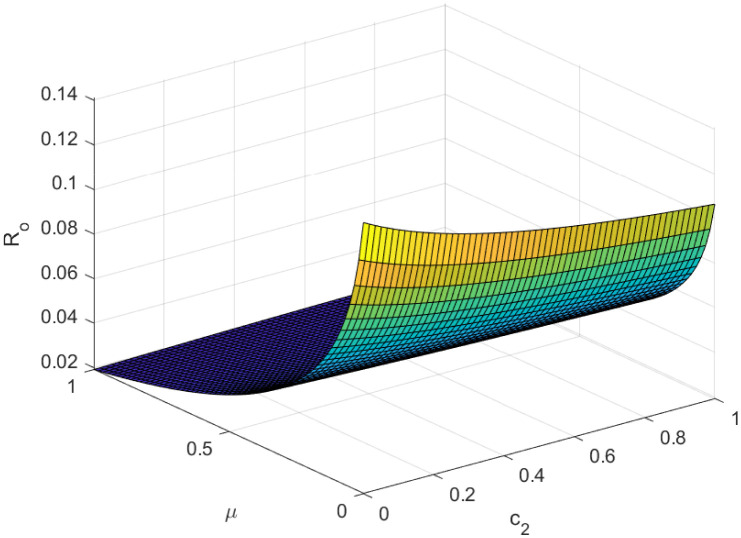
Effect of *μ* and *c*_2_ on reproductive number.

**Fig 3:**
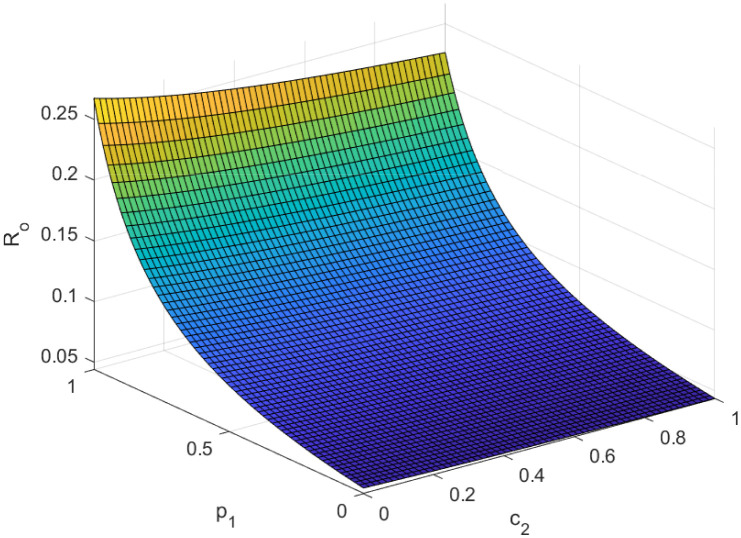
Effect of *p*_1_ and *c*_2_ on reproductive number.

**Fig 4:**
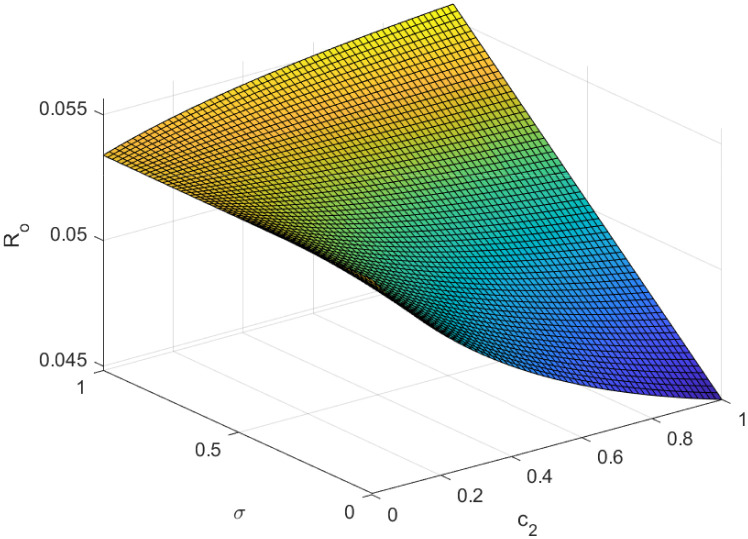
Effect of *σ* and *c*_2_ on reproductive number.

**Fig 5:**
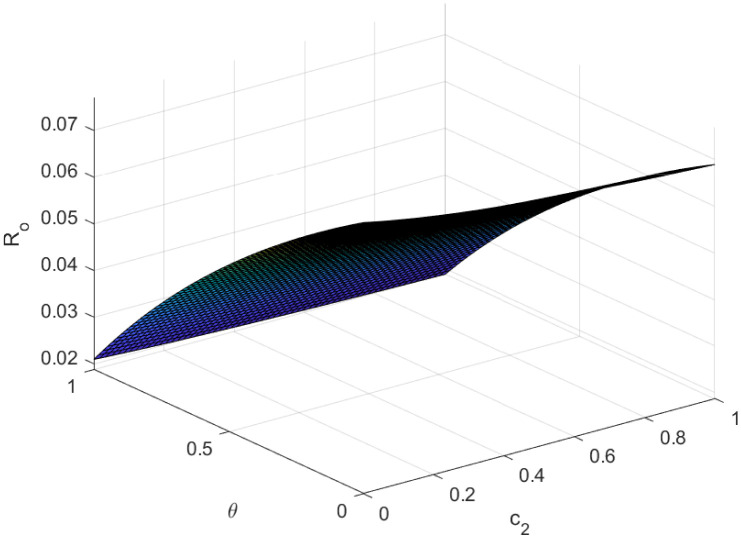
Effect of *θ* and *c*_2_ on reproductive number.

**Fig 6:**
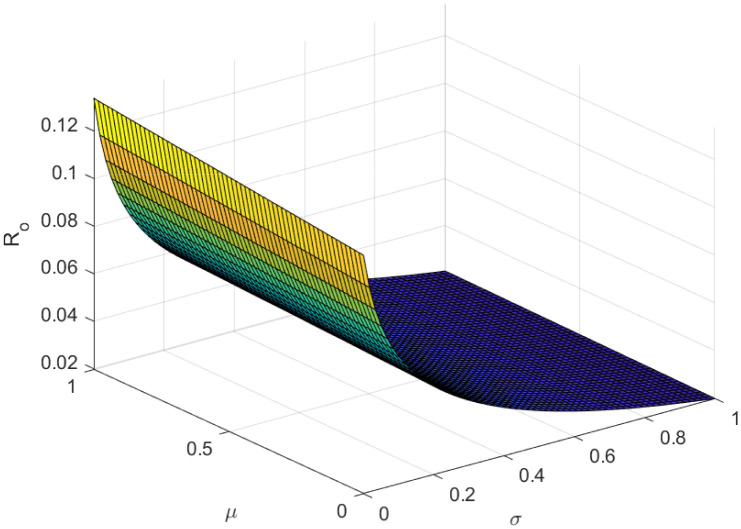
Effect of *μ* and *σ* on reproductive number.

Fundamentally, the fundamental reproduction number is related to both the disease’s propagation and containment. The disease’s spread and containment are shown by this number. If this threshold amount *R*_0_ < 1, the disease then vanishes from the population. Implementing preventive measures aids in managing the spread of an epidemic. However, if *R*_0_ > 1 illness the form of an epidemic and becomes a lifelong fixture in society. Figs 2–6 illustrate the effects of various parameters on reproduction number for biological viability and disease control.

**Theorem 3.1** Let Ψ denote a rectangular region, model (8) with initial values S(0)>0, E(0)>0, A(0)>0, C(0)>0, T(0)>0, R(0)>0, exist, bounded, positively invariant and attracting for all *t* > 0.

**Proof.** We show that $\frac{\partial {ϒ}_{j}}{\partial y_{j}},j=1,2,\ldots ,6$ are continuous and bounded in Ω.

Let $A_{1}=\mu -\mu p_{1}C-\mu \theta R+u_{2}R-\left(c\omega \left(A+\gamma C\right)+\left(\mu +\tau \right)\right)S$ take a derivative with regard to the state variable from the first system of Eq (8) gives us:

$$\begin{array}{c} \frac{\partial {ϒ}_{1}}{\partial S}=-\left(c\omega \left(A+\gamma C\right)+\left(\mu +\tau \right)\right),|\frac{\partial {ϒ}_{1}}{\partial S}|=|-(c\omega (A+\gamma C)+(\mu +\tau ))|<\infty , \end{array}$$

$$\begin{array}{c} \frac{\partial {ϒ}_{1}}{\partial E}=0,|\frac{\partial {ϒ}_{1}}{\partial E}|=0<\infty ,\frac{\partial {ϒ}_{1}}{\partial A}=-c\omega S,|\frac{\partial {ϒ}_{1}}{\partial A}|=|-c\omega S|<\infty , \end{array}$$

$$\begin{array}{c} \frac{\partial {ϒ}_{1}}{\partial C}=-\mu p_{1}-c\omega \gamma S,|\frac{\partial {ϒ}_{1}}{\partial C}|=|-\mu p_{1}-c\omega \gamma S|<\infty , \end{array}$$

$$\begin{array}{c} \frac{\partial {ϒ}_{1}}{\partial T}=0,|\frac{\partial {ϒ}_{1}}{\partial T}|=0<\infty ,\frac{\partial {ϒ}_{1}}{\partial R}=-\mu \theta +u_{2},|\frac{\partial {ϒ}_{1}}{\partial R}|=|-\mu \theta +u_{2}|<\infty . \end{array}$$

The finite and bounded partial derivatives of the entire system of equations exist.

**Theorem 3.2** Given S≥0, E≥0, A≥0, C≥0, T≥0, R≥0. Then the solution (S(t),E(t),A(t),C(t),T(t),R(t)) of model (8) are positive for all *t* > 0.

**Proof.** From model (8), we have

MABCDtνS|S=0=μ-μp1C-μθR+u2R≥0,MABCDtνE|E=0=cω(A+γC)S≥0,MABCDtνA|A=0=c1E≥0,MABCDtνC|C=0=c2E+δ1A≥0,MABCDtνT|T=0=σC≥0,MABCDtνR|R=0=ϕT+δ2A+τS≥0, (11)

which suggests that under non-negative initial circumstances, S(t),E(t),A(t),C(t),T(t) and R(t) are all non-negative.

### 3.2 Properties of modified Atangana-Baleanu fractional operator

Through the following, we demonstrate that there is a nonzero solution to the homogeneous fractional initial value problem. The following established formulas will be employed:

$$\begin{array}{c} L(E_{\nu }\left(℘t^{\nu }\right))=\frac{s^{\nu -1}}{s^{\nu }-℘},\;|\frac{℘}{s^{\nu }}|<1, \end{array}$$ (12)

$$\begin{array}{c} L(t^{\nu -1}E_{\nu ,\nu }\left(℘t^{\nu }\right))=\frac{1}{s^{\nu }-℘},\;|\frac{℘}{s^{\nu }}|<1. \end{array}$$ (13)

**Lemma 3.1** [20] Consider the fractional initial value problem

{MABCDtνN(t)=ΦN(t),N(0)=N0, (14)

where

$$\begin{array}{c} N\left(t\right)=\{\begin{array}{c} S(t)\\ E(t)\\ A(t)\\ C(t)\\ T(t)\\ R(t) \end{array},\;\;N\left(0\right)=\{\begin{array}{c} S(0)\\ E(0)\\ A(0)\\ C(0)\\ T(0)\\ R(0) \end{array}, \end{array}$$ (15)

and 0 < *ν* < 1.

**(1)** For $\Phi =\frac{B(\nu )}{1-\nu }$, the solution is given by

$$\begin{array}{c} \begin{array}{c} N\left(t\right)=N_{0}\{\begin{array}{c} -\frac{t^{-\nu }}{\mu_{\nu }\Gamma \left(1-\nu \right)},\;t\neq 0,\\ 1,\;t=0. \end{array} \end{array} \end{array}$$

**(2)** For $\Phi \neq \frac{B(\nu )}{1-\nu }$, the solution is given by

$$\begin{array}{c} \begin{array}{c} N\left(t\right)=N_{0}\{\begin{array}{c} \frac{E_{\nu }(\mu_{\nu }\frac{\chi_{\nu }}{1-\chi_{\nu }})}{1-\chi_{\nu }},\;t\neq 0\\ 1,\;t=0 \end{array} \end{array} \end{array}$$

where $\chi_{\nu }=\frac{\Phi (1-\nu )}{B(\nu )}$.

**Proof**.

**(1)** Given that

$$\begin{array}{c} \int_{0}^{t}{\left(t-\vartheta \right)}^{\nu -1}E_{\nu ,\nu }\left(-\mu_{\nu }{\left(t-\vartheta \right)}^{\nu }\right)\vartheta^{-\nu }d\vartheta =\Gamma \left(1-\nu \right)E_{\nu }(-\mu_{\nu }t^{\nu }), \end{array}$$ (16)

we have for *t* > 0,

MABCD0νN(t)=B(ν)1-ν(N(t)-Eν(-μνtν)N0-μν∫0t(t-ϑ)ν-1Eν,ν(-μν(t-ϑ)ν)ϑ-ν×(-N0μνΓ(1-ν))dϑ)=B(ν)1-ν(N(t)-Eν(-μνtν)N0+Eν(-μνtν)N0)=ΦN(t),

which completes the proof.

**(2)** Using Eqs (12) and (13) for *t* > 0,

L{MABCD0νN,s}=B(ν)1-ν1sν+με(S0sν1-χν×sν-1sν-μνχν1-χν-S0sν-1)=B(ν)1-νN0χν1-χνsν-1sν-μνχν1-χν

$$\begin{array}{c} =\Phi \frac{N_{0}}{1-\chi_{\nu }}\frac{s^{\nu -1}}{s^{\nu }-\mu_{\nu }\frac{\chi_{\nu }}{1-\chi_{\nu }}}=\Phi \frac{N_{0}}{1-\chi_{\nu }}L(E_{\nu }(\mu_{\nu }\frac{\chi_{\nu }}{1-\chi_{\nu }}t^{\nu })), \end{array}$$

which completes the proof.

**Lemma 3.2** [20] Consider the linear fractional differential equation

{MABCD0νN(t)+ΦN=Ji(t),t>0,N(0)=N0,i=1,2,…,6.

For 0 < *ν* < 1, and $\Phi \neq \frac{B(\nu )}{1-\nu }$, the fractional initial value problem mentioned above has a solution provided by

$$\begin{array}{c} \begin{array}{c} N\left(t\right)=\{\begin{array}{c} N^{*},\;t\neq 0,\\ N_{0},\;t=0, \end{array} \end{array} \end{array}$$ (17)

where

$$N^{*}=N_{0}\frac{B(\nu )}{\ell_{\nu }}E_{\nu }(-\frac{\Phi }{\ell_{\nu }}t^{\nu })+\frac{1-\nu }{\ell_{\nu }}J_{1}\left(t\right)+\frac{1-\nu }{\ell_{\nu }}(\mu_{\nu }-\frac{\Phi }{\ell_{\nu }})(t^{\nu -1}E_{\nu ,\nu }(-\frac{\Phi }{\ell_{\nu }}t^{\nu }))J_{i},$$

*i* = 1, 2, …, 6 and $\ell_{\nu }=B\left(\nu \right)+\Phi \left(1-\nu \right).$

**Proof** It is simple to confirm that by using Eqs (12) and (13).

$$\begin{array}{c} L(N,s)=\frac{N_{0}B\left(\nu \right)s^{\nu -1}+\left(1-\nu \right)\left(s^{\nu }+\mu_{\nu }\right)L\left(J_{i},s\right)}{\ell_{\nu }s^{\nu }+\Phi \nu }. \end{array}$$ (18)

By Eq (5) we have

$$\begin{array}{c} \begin{array}{c} L{(}^{MABC}D_{0}^{\nu }N+\Phi N,s)=\frac{B(\nu )}{1-\nu }\frac{s^{\nu }L(N^{*},s)-s^{\nu -1}v_{0}}{s^{\nu }+\mu_{\nu }}+\Phi L(N,s) \end{array}. \end{array}$$ (19)

Simple calculations will yield

$$\begin{array}{c} \begin{array}{c} L{(}^{MABC}D_{0}^{\nu }N+\Phi N,s)=\frac{1}{\left(1-\nu \right)\left(s^{\nu }+\mu_{\nu }\right)}((\ell_{\nu }s^{\alpha }+\Phi \nu )L(N^{*},s)-B\left(\nu \right)s^{\nu -1}N_{0}), \end{array}. \end{array}$$ (20)

By putting Eq (18) into Eq (20), the following outcome is obtained:

$$\begin{array}{c} \;\begin{array}{c} L{(}^{MABC}D_{0}^{\nu }N+\Phi N,s)=\frac{1}{\left(1-\nu \right)\left(s^{\nu }+\mu_{\nu }\right)}(B\left(\nu \right)s^{\nu -1}N_{0}+\left(1-\nu \right)\left(s^{\nu }+\mu_{\nu }\right)L\left(J_{i},s\right)-B\left(\nu \right)s^{\nu -1}N_{0}), \end{array} \end{array}$$

$$\begin{array}{c} =L(J_{i},s),\;i=1,2,\ldots 6, \end{array}$$

and the proof is complete.

## 4 Global stability

In this section, we discussed global stability through the concept of Lyapunov function. **Theorem 4.1** The global asymptotic stability of the endemic equilibrium points E* of the HBV model is achieved when the reproductive number *R*_0_ > 0.

**Proof.** The Lyapunov function is defined by

$$\begin{array}{c} \begin{array}{cc} L(S^{*},E^{*},A^{*},C^{*},T^{*},R^{*}) & =(S-S^{*}-S^{*}\text{ln}\frac{S}{S^{*}})+(E-E^{*}-E^{*}\text{ln}\frac{E}{E^{*}})\\ & +(A-A^{*}-A^{*}\text{ln}\frac{A}{A^{*}})+(C-C^{*}-C^{*}\text{ln}\frac{C}{C^{*}})\\ & +(T-T^{*}-T^{*}\text{ln}\frac{T}{T^{*}})+(R-R^{*}-R^{*}\text{ln}\frac{R}{R^{*}}). \end{array} \end{array}$$ (21)

As a result, using the derivative with regard to *t*

MABCDtνL≤(S-S*S)S′+(E-E*E)E′+(A-A*A)A′+(C-C*C)C′+(T-T*T)T′+(R-R*R)R′. (22)

Their derivative values can now be expressed as follows:

MABCDtνL≤(S-S*S)(μ-μp1C-μθR+u2R-(cω(A+γC)+m1)S)+(E-E*E)(cω(A+γC)S-m2E)+(A-A*A)(c1E-m3A)+(C-C*C)(c2E+δ1A-(m4-μp1)C)+(T-T*T)(σC-m5T)+(R-R*R)(ϕT+δ2A+τS+(μθ-m6)R). (23)

Putting $S=S-S^{*}$, $E=E-E^{*}$, $A=A-A^{*}$, $C=C-C^{*}$, $T=T-T^{*}$ and $R=R-R^{*}$ leads to

MABCDtνL≤(S-S*S)(μ-μp1(C-C*)-μθ(R-R*)+u2(R-R*)-(cω((A-A*)+γ(C-C*)+m1)(S-S*))+(E-E*E)(cω((A-A*)+γ(C-C*))(S-S*)-m2(E-E*))+(A-A*A)(c1(E-E*)-m3(A-A*))+(C-C*C)(c2(E-E*)+δ1(A-A*)-(m4-μp1)(C-C*))+(T-T*T)(σ(C-C*)-m5(T-T*))+(R-R*R)(ϕ(T-T*)+δ2(A-A*)+τ(S-S*)+(μθ-m6)(R-R*)). (24)

We can change the above as follows

MABCDtνL≤μ-μS*S-μp1C+μp1CS*S+μp1C*-μp1C*S*S-μθR+μθRS*S+μθR*-μθR*S*S+u2R-u2RS*S-u2R*+u2R*S*S-l1+l2+cωAS-cωASE*E-cωAS*+cωAS*E*E-cωA*S-cωA*SE*E+cωA*S*-cωA*S*E*E+cωγCS-cωγCSE*E-cωγCS*+cωγCS*E*E-cωγC*S+cωγC*SE*E+cωγC*S*-cωγC*S*E*E+c1E-c1EA*A-c1E*+c1E*A*A+c2E-c2EC*C-c2E*+c2E*C*C+δ1A-δ1AC*C-δ1A*+δ1A*C*C+σC-σCT*T-σC*+σC*T*T+ϕT-ϕTR*R-ϕT*+ϕT*R*R+δ2A-δ2AR*R-δ2A*+δ2A*R*R+τS-τSR*R-τS*+τS*R*R, (25)

where

$$\begin{array}{c} \begin{array}{cc} l_{1} & =c\omega A\frac{{\left(S-S^{*}\right)}^{2}}{S}+c\omega \gamma C\frac{{\left(S-S^{*}\right)}^{2}}{S}+c_{1}\frac{{\left(E-E^{*}\right)}^{2}}{E}+c_{2}\mu \frac{{\left(E-E^{*}\right)}^{2}}{E}+\delta_{1}\frac{{\left(A-A^{*}\right)}^{2}}{A}\\ & +\delta_{2}\frac{{\left(A-A^{*}\right)}^{2}}{A}+\mu \frac{{\left(A-A^{*}\right)}^{2}}{A}+\sigma \frac{{\left(C-C^{*}\right)}^{2}}{C}+d\frac{{\left(C-C^{*}\right)}^{2}}{C}+\mu \frac{{\left(C-C^{*}\right)}^{2}}{C}+\phi \frac{{\left(T-T^{*}\right)}^{2}}{T}\\ & +\mu \frac{{\left(T-T^{*}\right)}^{2}}{T}+\mu \frac{{\left(R-R^{*}\right)}^{2}}{R}+u_{2}\frac{{\left(R-R^{*}\right)}^{2}}{R}, \end{array} \end{array}$$ (26)

$$\begin{array}{c} \begin{array}{cc} l_{2} & =c\omega A^{*}\frac{{\left(S-S^{*}\right)}^{2}}{S}+c\omega \gamma C^{*}\frac{{\left(S-S^{*}\right)}^{2}}{S}+\mu \frac{{\left(S-S^{*}\right)}^{2}}{S}+\tau \frac{{\left(S-S^{*}\right)}^{2}}{S}+\mu p_{1}\frac{{\left(C-C^{*}\right)}^{2}}{C}\\ & +\mu \theta \frac{{\left(R-R^{*}\right)}^{2}}{R}. \end{array} \end{array}$$ (27)

To simplify things, the above can be written as

MABCDtνL=Ω1-Ω2. (28)

where

$$\begin{array}{c} \begin{array}{cc} \Omega_{1} & =\mu +\mu p_{1}C\frac{S^{*}}{S}+\mu p_{1}C^{*}+\mu \theta R\frac{S^{*}}{S}+\mu \theta R^{*}+u_{2}R+u_{2}R^{*}\frac{S^{*}}{S}+l_{2}+c\omega AS+c\omega AS^{*}\frac{E^{*}}{E}\\ & +c\omega SA^{*}\frac{E^{*}}{E}+c\omega A^{*}S^{*}+c\omega \gamma CS+c\omega \gamma CS^{*}\frac{E^{*}}{E}+c\omega \gamma C^{*}S\frac{E^{*}}{E}+c\omega \gamma C^{*}S^{*}+c_{1}E\\ & +c_{1}E^{*}\frac{A^{*}}{A}+c_{2}E+c_{2}E^{*}\frac{C^{*}}{C}+\delta_{1}A+\delta_{1}A^{*}\frac{C^{*}}{C}+\sigma C+\sigma C^{*}\frac{T^{*}}{T}+\phi T+\phi T^{*}\frac{R^{*}}{R}\\ & +\delta_{2}A+\delta_{2}A^{*}\frac{R^{*}}{R}+\tau S+\tau S^{*}\frac{R^{*}}{R}, \end{array} \end{array}$$ (29)

and

$$\begin{array}{c} \begin{array}{cc} \Omega_{2} & =\mu \frac{S^{*}}{S}+\mu p_{1}C+\mu p_{1}C^{*}\frac{S^{*}}{S}+\mu \theta R+\mu \theta R^{*}\frac{S^{*}}{S}+u_{2}R\frac{S^{*}}{S}+u_{2}R^{*}+l_{1}+c\omega AS\frac{E^{*}}{E}\\ & +c\omega AS^{*}+c\omega A^{*}S+c\omega A^{*}S^{*}\frac{E^{*}}{E}+c\omega \gamma CS\frac{E^{*}}{E}+c\omega \gamma CS^{*}+c\omega \gamma C^{*}S+c\omega \gamma C^{*}S^{*}\frac{E^{*}}{E}\\ & +c_{1}E\frac{A^{*}}{A}+c_{1}E^{*}+c_{2}E\frac{C^{*}}{C}+c_{2}E^{*}+\delta_{1}A\frac{C^{*}}{C}+\delta_{1}A^{*}+\sigma C\frac{T^{*}}{T}+\sigma C^{*}+\phi T\frac{R^{*}}{R}+\phi T^{*}\\ & +\delta_{2}A\frac{R^{*}}{R}+\delta_{2}A^{*}+\tau S\frac{R^{*}}{R}+\tau S^{*}. \end{array} \end{array}$$ (30)

It has been concluded that if Ω_1_ < Ω_2_, this yields MABCDtνL<0, when, however $S=S^{*}$, $E=E^{*}$, $A=A^{*}$, $C=C^{*}$, $T=T^{*}$, $R=R^{*}$ is

0=Ω1-Ω2⇒MABCDtνL=0, (31)

we can see that the suggested model has the biggest compact invariant set in

{(S*,E*,A*,C*,T*,R*)∈Γ:MABCDtνL=0}, (32)

the suggested model’s endemic equilibrium is represented by point E_*_. It follows from the Lasalles invariant idea that E_*_ is globally asymptotic and stable in Γ if Ω_1_ < Ω_2_.

## 5 Existence criteria

By utilizing the consecutive iterative approach, we will be able to demonstrate the existence results of the modified ABC-fractional order HBV model (8). Using the integral from definition 2.4 and model (8) with the aid of Lemma 2.1 yields

$$\begin{array}{c} \begin{array}{cc} S(t) & =S\left(0\right)+\frac{1-\nu }{B(\nu )}\left[\mu -\mu p_{1}C-\mu \theta R+u_{2}R-\left(c\omega \left(A+\gamma C\right)+m_{1}\right)\right]\\ & +\frac{\nu }{B(\nu )\Gamma (\nu )}\int_{0}^{t}{\left(t-\vartheta \right)}^{\nu -1}\left[\mu -\mu p_{1}C-\mu \theta R+u_{2}R-\left(c\omega \left(A+\gamma C\right)+m_{1}\right)\right]d\vartheta \\ & -\frac{1-\nu }{B(\nu )}\left[\mu -\mu p_{1}C^{0}-\mu \theta R^{0}+u_{2}R^{0}-\left(c\omega \left(A^{0}+\gamma C^{0}\right)+m_{1}\right)\right](1+\frac{\gamma_{\nu }}{\Gamma (\nu +1)}t^{\nu }), \end{array} \end{array}$$ (33)

$$\begin{array}{c} \;\begin{array}{cc} E(t) & =E\left(0\right)+\frac{1-\nu }{B(\nu )}\left[c\omega \left(A+\gamma C\right)S-m_{2}E\right]+\frac{\nu }{B(\nu )\Gamma (\nu )}\int_{0}^{t}{\left(t-\vartheta \right)}^{\nu -1}\left[c\omega \left(A+\gamma C\right)S-m_{2}E\right]d\vartheta \\ & -\frac{1-\nu }{B(\nu )}\left[c\omega \left(A^{0}+\gamma C^{0}\right)S^{0}-m_{2}E^{0}\right](1+\frac{\gamma_{\nu }}{\Gamma (\nu +1)}t^{\nu }), \end{array} \end{array}$$ (34)

$$\begin{array}{c} \begin{array}{cc} A(t) & =A\left(0\right)+\frac{1-\nu }{B(\nu )}\left[c_{1}E-m_{3}A\right]+\frac{\nu }{B(\nu )\Gamma (\nu )}\int_{0}^{t}{\left(t-\vartheta \right)}^{\nu -1}\left[c_{1}E-m_{3}A\right]d\vartheta \\ & -\frac{1-\nu }{B(\nu )}\left[c_{1}E^{0}-m_{3}A^{0}\right](1+\frac{\gamma_{\nu }}{\Gamma (\nu +1)}t^{\nu }), \end{array} \end{array}$$ (35)

$$\begin{array}{c} \begin{array}{cc} C(t) & =C\left(0\right)+\frac{1-\nu }{B(\nu )}\left[c_{2}E+\delta_{1}A-\left(m_{4}-\mu p_{1}\right)C\right]\\ & +\frac{\nu }{B(\nu )\Gamma (\nu )}\int_{0}^{t}{\left(t-\vartheta \right)}^{\nu -1}\left[c_{2}E+\delta_{1}A-\left(m_{4}-\mu p_{1}\right)C\right]d\vartheta \\ & -\frac{1-\nu }{B(\nu )}\left[c_{2}E^{0}+\delta_{1}A-\left(m_{4}-\mu p_{1}\right)C^{0}\right](1+\frac{\gamma_{\nu }}{\Gamma (\nu +1)}t^{\nu }), \end{array} \end{array}$$ (36)

$$\begin{array}{c} \begin{array}{cc} T(t) & =T\left(0\right)+\frac{1-\nu }{B(\nu )}\left[\sigma C-m_{5}T\right]+\frac{\nu }{B(\nu )\Gamma (\nu )}\int_{0}^{t}{\left(t-\vartheta \right)}^{\nu -1}\left[\sigma C-m_{5}T\right]d\vartheta \\ & -\frac{1-\nu }{B(\nu )}\left[\sigma C^{0}-m_{5}T^{0}\right](1+\frac{\gamma_{\nu }}{\Gamma (\nu +1)}t^{\nu }), \end{array} \end{array}$$ (37)

$$\begin{array}{c} \begin{array}{cc} R(t) & =R\left(0\right)+\frac{1-\nu }{B(\nu )}\left[\phi T+\delta_{2}A+\tau S+\left(\mu \theta -m_{6}\right)R\right]\\ & +\frac{\nu }{B(\nu )\Gamma (\nu )}\int_{0}^{t}{\left(t-\vartheta \right)}^{\nu -1}\left[\phi T+\delta_{2}A+\tau S+\left(\mu \theta -m_{6}\right)R\right]d\vartheta \\ & -\frac{1-\nu }{B(\nu )}\left[\phi T^{0}+\delta_{2}A^{0}+\tau S^{0}+\left(\mu \theta -m_{6}\right)R^{0}\right](1+\frac{\gamma_{\nu }}{\Gamma (\nu +1)}t^{\nu }), \end{array} \end{array}$$ (38)

To make the calculations easier, we define *κ*_*i*_ for *j* = 1, 2, 3, 4, 5, 6, as shown below

$$\begin{array}{c} \kappa_{1}(t,\;S(t))=\mu -\mu p_{1}C-\mu \theta R+u_{2}R-\left(c\omega \left(A+\gamma C\right)+m_{1}\right)S, \end{array}$$

$$\begin{array}{c} \kappa_{2}(t,\;E(t))=c\omega \left(A+\gamma C\right)S-m_{2}E, \end{array}$$

$$\begin{array}{c} \kappa_{3}(t,A(t))=c_{1}E-m_{3}A, \end{array}$$

$$\begin{array}{c} \kappa_{4}(t,C(t))=c_{2}E+\delta_{1}A-\left(m_{4}-\mu p_{1}\right)C, \end{array}$$

$$\kappa_{5}\left(t,A\left(t\right)\right)=\sigma \mathbb{C}-m_{5}T,$$

$$\begin{array}{c} \kappa_{6}(t,A(t))=\phi T+\delta_{2}A+\tau S+\left(\mu \theta -m_{6}\right)R. \end{array}$$

The modified ABC model is presumptively solved, with the following assumption:

(H*) Let us consider $S,\bar{S},E,\bar{E},A,\bar{A},C,\bar{C},T,\bar{T},R,\bar{R}\in L[0,1],$ be continuous functions with an upper limit denoted by $∥S∥\leq \varphi_{1},$ $∥E∥\leq \varphi_{2},$ $∥A∥\leq \varphi_{3},$ $∥C∥\leq \varphi_{4},$ $∥T∥\leq \varphi_{5},$ $∥R∥\leq \varphi_{6},$ where *φ*_1_, *φ*_2_, *φ*_3_, *φ*_4_, *φ*_5_, *φ*_6_ are all positive constants. Furthermore, we suppose that: $\varsigma_{1}=c\omega \varphi_{3}+\gamma \varphi_{4}+m_{1},$ $\varsigma_{2}=m_{2},$ $\varsigma_{3}=m_{3},$ $\varsigma_{4}=m_{4}-\mu p_{1},$ $\varsigma_{5}=m_{5},$ $\varsigma_{6}=\left(\mu \theta +m_{6}\right).$

**Theorem 5.1** If the presumption (H*) holds true and all the $\varsigma_{j}<1$ for $j\in N_{1}^{6}$ the *κ*_*j*_ for $j\in N_{1}^{6}$ satisfying the Lipschitz condition.

**Proof.** We begin by showing that the Lipschitz condition is satisfied for $\kappa_{1}\left(t,S\right)$. To do this, we use the implication of (H*)

$$\begin{array}{c} \begin{array}{cc} ∥\kappa_{1}\left(t,S\right)-\kappa_{1}(t,\bar{S}∥ & =∥(\mu -\mu p_{1}C-\mu \theta R+u_{2}R-\left(c\omega \left(A+\gamma C\right)+m_{1}\right)S)\\ & -(\mu -\mu p_{1}C-\mu \theta R+u_{2}R-\left(c\omega \left(A+\gamma C\right)+m_{1}\right)\bar{S})∥\\ & \leq c\omega ∥S-\bar{S}∥∥A∥+\gamma ∥S-\bar{S}∥∥C∥+m_{1}∥S-\bar{S}∥\\ & \leq \left(c\omega \varphi_{3}+\gamma \varphi_{4}+m_{1}\right)∥S-\bar{S}∥\\ & =\varsigma_{1}∥S-\bar{S}∥ \end{array} \end{array}$$ (39)

where $\varsigma_{1}=c\omega \varphi_{3}+\gamma \varphi_{4}+m_{1}.$ Hence, *κ*_1_ fulfills the Lipschitz condition with constant *φ*_1_. Next, for $\kappa_{2}\left(t,E\right),$ consider

$$\begin{array}{c} \begin{array}{cc} ∥\kappa_{2}\left(t,E\right)-\kappa_{2}\left(t,\bar{E}\right)∥ & =∥\left(c\omega \left(A+\gamma C\right)S-m_{2}E\right)-\left(c\omega \left(A+\gamma C\right)S-m_{2}\bar{E}\right)∥\\ & \leq m_{2}∥E-\bar{E}∥=\varsigma_{2}∥E-\bar{E}∥, \end{array} \end{array}$$ (40)

where $\varsigma_{2}=m_{2}.$ Hence, *κ*_2_ salsify the Lipschitz condition with *φ*_2_. Similarly, for $\kappa_{3}\left(t,A\right),$ we have

$$\begin{array}{c} \begin{array}{cc} ∥\kappa_{3}\left(t,A\right)-\kappa_{3}\left(t,\bar{A}\right)∥ & =∥\left(c_{1}E-m_{3}A\right)-\left(c_{1}E-m_{3}\bar{A}\right)∥\\ & \leq m_{3}∥A-\bar{A}∥=\varsigma_{3}∥A-\bar{A}∥, \end{array} \end{array}$$ (41)

where $\varsigma_{3}=m_{3}.$ Thus, *κ*_3_ fulfills the Lipschitz condition with *φ*_3_. For $\kappa_{4}\left(t,C\right),$ we also have

$$\begin{array}{c} \begin{array}{cc} ∥\kappa_{2}\left(t,C\right)-\kappa_{2}\left(t,\bar{C}\right)∥ & =∥\left(c_{2}E+\delta_{1}A-\left(m_{4}-\mu p_{1}\right)C\right)-\left(c_{2}E+\delta_{1}A-\left(m_{4}-\mu p_{1}\right)\bar{C}\right)∥\\ & \leq \left(m_{4}-\mu p_{1}\right)∥C-\bar{C}∥=\varsigma_{4}∥C-\bar{C}∥, \end{array} \end{array}$$ (42)

where $\varsigma_{4}=m_{4}-\mu p_{1}.$ Hence, *κ*_3_ fulfills the Lipschitz condition with *φ*_3_. Similarly, for $\kappa_{5}\left(t,T\right),$ consider

$$\begin{array}{c} \begin{array}{cc} ∥\kappa_{5}\left(t,T\right)-\kappa_{5}\left(t,\bar{T}\right)∥ & =∥\left(\sigma C-m_{5}T\right)-\left(\sigma C-m_{5}\bar{T}\right)∥\\ & \leq m_{5}∥T-\bar{T}∥=\varsigma_{5}∥T-\bar{T}∥, \end{array} \end{array}$$ (43)

where $\varsigma_{5}=m_{5}.$ Thus, *κ*_5_ fulfills the Lipschitz condition with constant *φ*_5_. For $\kappa_{6}\left(t,C\right),$ we also have

$$\begin{array}{c} \begin{array}{cc} ∥\kappa_{6}\left(t,R\right)-\kappa_{6}\left(t,\bar{R}\right)∥ & =∥\left(\varphi T+\delta_{2}A+\tau S+\left(\mu \theta -m_{6}\right)R\right)-\left(\varphi T+\delta_{2}A+\tau S+\left(\mu \theta -m_{6}\right)\bar{R}\right)∥\\ & \leq \left(\mu \theta +m_{6}\right)∥R-\bar{R}∥=\varsigma_{6}∥R-\bar{R}∥, \end{array} \end{array}$$ (44)

where $\varsigma_{6}=\left(\mu \theta +m_{6}\right).$ With constant *φ*_6_, *κ*_5_ likewise meets the Lipschitz requirement. Therefore, all of the *κ*_*j*_ for *j* = 1, 2, 3, 4, 5, 6, from (39)–44) fulfill the Lipschitz condition and are therefore proved. Assume for now

$$\begin{array}{c} \begin{array}{cc} S(t) & =S\left(0\right)+\frac{1-\nu }{B(\nu )}\kappa_{1}(t,S(t))+\frac{\nu }{B(\nu )\Gamma (\nu )}\int_{0}^{t}{\left(t-\vartheta \right)}^{\nu -1}\kappa_{1}(\vartheta ,S(\vartheta ))d\vartheta \\ & -\frac{1-\nu }{B(\nu )}\kappa_{1}^{0}(t,S(t))(1+\frac{\gamma_{\nu }}{\Gamma (\nu +1)}t^{\nu }), \end{array} \end{array}$$ (45)

$$\begin{array}{c} \begin{array}{cc} E(t) & =E\left(0\right)+\frac{1-\nu }{B(\nu )}\kappa_{2}(t,E(t))+\frac{\nu }{B(\nu )\Gamma (\nu )}\int_{0}^{t}{\left(t-\vartheta \right)}^{\nu -1}\kappa_{2}(\vartheta ,E(\vartheta ))d\vartheta \\ & -\frac{1-\nu }{B(\nu )}\kappa_{2}^{0}(t,E(t))(1+\frac{\gamma_{\nu }}{\Gamma (\nu +1)}t^{\nu }), \end{array} \end{array}$$ (46)

$$\begin{array}{c} \begin{array}{cc} A(t) & =A\left(0\right)+\frac{1-\nu }{B(\nu )}\kappa_{3}(t,A(t))+\frac{\nu }{B(\nu )\Gamma (\nu )}\int_{0}^{t}{\left(t-\vartheta \right)}^{\nu -1}\kappa_{3}(\vartheta ,A(\vartheta ))d\vartheta \\ & -\frac{1-\nu }{B(\nu )}\kappa_{3}^{0}(t,A(t))(1+\frac{\gamma_{\nu }}{\Gamma (\nu +1)}t^{\nu }), \end{array} \end{array}$$ (47)

$$\begin{array}{c} \begin{array}{cc} C(t) & =C\left(0\right)+\frac{1-\nu }{B(\nu )}\kappa_{4}(t,C(t))+\frac{\nu }{B(\nu )\Gamma (\nu )}\int_{0}^{t}{\left(t-\vartheta \right)}^{\nu -1}\kappa_{4}(\vartheta ,S(\vartheta ))d\vartheta \\ & -\frac{1-\nu }{B(\nu )}\kappa_{4}^{0}(t,C(t))(1+\frac{\gamma_{\nu }}{\Gamma (\nu +1)}t^{\nu }), \end{array} \end{array}$$ (48)

$$\begin{array}{c} \begin{array}{cc} T(t) & =T\left(0\right)+\frac{1-\nu }{B(\nu )}\kappa_{5}(t,T(t))+\frac{\nu }{B(\nu )\Gamma (\nu )}\int_{0}^{t}{\left(t-\vartheta \right)}^{\nu -1}\kappa_{5}(\vartheta ,T(\vartheta ))d\vartheta \\ & -\frac{1-\nu }{B(\nu )}\kappa_{5}^{0}(t,T(t))(1+\frac{\gamma_{\nu }}{\Gamma (\nu +1)}t^{\nu }), \end{array} \end{array}$$ (49)

$$\begin{array}{c} \begin{array}{cc} R(t) & =R\left(0\right)+\frac{1-\nu }{B(\nu )}\kappa_{6}(t,R(t))+\frac{\nu }{B(\nu )\Gamma (\nu )}\int_{0}^{t}{\left(t-\vartheta \right)}^{\nu -1}\kappa_{6}(\vartheta ,R(\vartheta ))d\vartheta \\ & -\frac{1-\nu }{B(\nu )}\kappa_{6}^{0}(t,R(t))(1+\frac{\gamma_{\nu }}{\Gamma (\nu +1)}t^{\nu }), \end{array} \end{array}$$ (50)

The recursive formulas for the model need to be defined next, and they are as follows:

$$\begin{array}{c} \begin{array}{cc} S_{n+1}\left(t\right)-S\left(0\right) & =\frac{1-\nu }{B(\nu )}\kappa_{1}(t,S_{n}(t))+\frac{\nu }{B(\nu )\Gamma (\nu )}\int_{0}^{t}{\left(t-\vartheta \right)}^{\nu -1}\kappa_{1}(\vartheta ,S_{n}(\vartheta ))d\vartheta \\ & -\frac{1-\nu }{B(\nu )}\kappa_{1}^{0}(t,S(t))(1+\frac{\gamma_{\nu }}{\Gamma (\nu +1)}t^{\nu }), \end{array} \end{array}$$ (51)

$$\begin{array}{c} \begin{array}{cc} E_{n+1}\left(t\right)-E\left(0\right) & =\frac{1-\nu }{B(\nu )}\kappa_{2}(t,E_{n}(t))+\frac{\nu }{B(\nu )\Gamma (\nu )}\int_{0}^{t}{\left(t-\vartheta \right)}^{\nu -1}\kappa_{2}(\vartheta ,E_{n}(\vartheta ))d\vartheta \\ & -\frac{1-\nu }{B(\nu )}\kappa_{2}^{0}(t,E(t))(1+\frac{\gamma_{\nu }}{\Gamma (\nu +1)}t^{\nu }), \end{array} \end{array}$$ (52)

$$\begin{array}{c} \begin{array}{cc} A_{n+1}\left(t\right)-A\left(0\right) & =\frac{1-\nu }{B(\nu )}\kappa_{3}(t,A_{n}(t))+\frac{\nu }{B(\nu )\Gamma (\nu )}\int_{0}^{t}{\left(t-\vartheta \right)}^{\nu -1}\kappa_{3}(\vartheta ,A_{n}(\vartheta ))d\vartheta \\ & -\frac{1-\nu }{B(\nu )}\kappa_{3}^{0}(t,A(t))(1+\frac{\gamma_{\nu }}{\Gamma (\nu +1)}t^{\nu }), \end{array} \end{array}$$ (53)

$$\begin{array}{c} \begin{array}{cc} C_{n+1}\left(t\right)-C\left(0\right) & =\frac{1-\nu }{B(\nu )}\kappa_{4}(t,C_{n}(t))+\frac{\nu }{B(\nu )\Gamma (\nu )}\int_{0}^{t}{\left(t-\vartheta \right)}^{\nu -1}\kappa_{4}(\vartheta ,C_{n}(\vartheta ))d\vartheta \\ & -\frac{1-\nu }{B(\nu )}\kappa_{4}^{0}(t,C(t))(1+\frac{\gamma_{\nu }}{\Gamma (\nu +1)}t^{\nu }), \end{array} \end{array}$$ (54)

$$\begin{array}{c} \begin{array}{cc} T_{n+1}\left(t\right)-T\left(0\right) & =\frac{1-\nu }{B(\nu )}\kappa_{5}(t,T_{n}(t))+\frac{\nu }{B(\nu )\Gamma (\nu )}\int_{0}^{t}{\left(t-\vartheta \right)}^{\nu -1}\kappa_{5}(\vartheta ,T_{n}(\vartheta ))d\vartheta \\ & -\frac{1-\nu }{B(\nu )}\kappa_{5}^{0}(t,T(t))(1+\frac{\gamma_{\nu }}{\Gamma (\nu +1)}t^{\nu }), \end{array} \end{array}$$ (55)

$$\begin{array}{c} \begin{array}{cc} R_{n+1}\left(t\right)-R\left(0\right) & =\frac{1-\nu }{B(\nu )}\kappa_{6}(t,R_{n}(t))+\frac{\nu }{B(\nu )\Gamma (\nu )}\int_{0}^{t}{\left(t-\vartheta \right)}^{\nu -1}\kappa_{6}(\vartheta ,R_{n}(\vartheta ))d\vartheta \\ & -\frac{1-\nu }{B(\nu )}\kappa_{6}^{0}(t,R(t))(1+\frac{\gamma_{\nu }}{\Gamma (\nu +1)}t^{\nu }), \end{array} \end{array}$$ (56)

**Theorem 5.2** If the following is true, then the modified ABC Hepatitis-B mathematical model (8) has a solution based on the supposition (H*):

$$\begin{array}{c} \Omega =\text{max}[\varphi_{1},\varphi_{2},\varphi_{3},\varphi_{4},\varphi_{5},\varphi_{6}]<1. \end{array}$$

**Proof.** We define six functions, which are as follows:

$$\begin{array}{c} \begin{array}{c} Z1_{n}\left(t\right)=S_{n+1}\left(t\right)-S\left(t\right),\\ Z2_{n}\left(t\right)=E_{n+1}\left(t\right)-E\left(t\right),\\ Z3_{n}\left(t\right)=A_{n+1}\left(t\right)-A\left(t\right),\\ Z4_{n}\left(t\right)=C_{n+1}\left(t\right)-C\left(t\right),\\ Z5_{n}\left(t\right)=T_{n+1}\left(t\right)-T\left(t\right),\\ Z6_{n}\left(t\right)=R_{n+1}\left(t\right)-R\left(t\right), \end{array} \end{array}$$

$$\begin{array}{cc} ∥Z1_{n}∥ & =∥\frac{1-\nu }{B(\nu )}\kappa_{1}(t,S_{n}(t))+\frac{\nu }{B(\nu )\Gamma (\nu )}\int_{0}^{t}{\left(t-\vartheta \right)}^{\nu -1}\kappa_{1}(\vartheta ,S_{n}(\vartheta ))d\vartheta \\ \; & -\frac{1-\nu }{B(\nu )}\kappa_{1}^{0}(t,S(t))(1+\frac{\gamma_{\nu }}{\Gamma (\nu +1)}t^{\nu })\\ \; & -[\frac{1-\nu }{B(\nu )}\kappa_{1}(t,S(t))+\frac{\nu }{B(\nu )\Gamma (\nu )}\int_{0}^{t}{\left(t-\vartheta \right)}^{\nu -1}\kappa_{1}(\vartheta ,S(\vartheta ))d\vartheta \\ \; & -\frac{1-\nu }{B(\nu )}\kappa_{1}^{0}(t,S(t))(1+\frac{\gamma_{\nu }}{\Gamma (\nu +1)}t^{\nu })]\\ \; & =\frac{\nu }{B(\nu )\Gamma (\nu )}\int_{0}^{t}{\left(t-\vartheta \right)}^{\nu -1}∥\kappa_{1}(\vartheta ,S_{n}(\vartheta ))-\kappa_{1}(\vartheta ,S(\vartheta ))∥d\vartheta \\ \; & +\frac{1-\nu }{B(\nu )}∥\kappa_{1}(t,S_{n}(t))-\kappa_{1}(t,S(t))∥\\ \; & \leq \frac{\nu }{B(\nu )\Gamma (\nu )}\int_{0}^{t}{\left(t-\vartheta \right)}^{\nu -1}\varphi_{1}∥S_{n-1}-S∥d\vartheta \\ \; & +\frac{1-\nu }{B(\nu )}\varphi_{1}∥S_{n-1}-S∥\\ \; & \leq [\frac{\nu }{B(\nu )\Gamma (\nu +1)}+\frac{1-\nu }{B(\nu )}]\varphi_{1}∥S_{n-1}-S∥\\ & \leq {\left[\frac{\nu }{B(\nu )\Gamma (\nu +1)}+\frac{1-\nu }{B(\nu )}\right]}^{n}\varphi_{1}^{n}∥S_{n-1}-S∥, \end{array}$$ (57)

where *φ* < 1 and *n* → ∞, *S*_*n*_ → *S* similarly we have

$$\begin{array}{c} ∥Z2_{n}∥\leq {\left[\frac{\nu }{B(\nu )\Gamma (\nu +1)}+\frac{1-\nu }{B(\nu )}\right]}^{n}\varphi_{2}^{n}∥E_{n-1}-E∥ \end{array}$$ (58)

$$\begin{array}{c} ∥Z3_{n}∥\leq {\left[\frac{\nu }{B(\nu )\Gamma (\nu +1)}+\frac{1-\nu }{B(\nu )}\right]}^{n}\varphi_{3}^{n}∥A_{n-1}-A∥ \end{array}$$ (59)

$$\begin{array}{c} ∥Z4_{n}∥\leq {\left[\frac{\nu }{B(\nu )\Gamma (\nu +1)}+\frac{1-\nu }{B(\nu )}\right]}^{n}\varphi_{4}^{n}∥C_{n-1}-C∥ \end{array}$$ (60)

$$\begin{array}{c} ∥Z5_{n}∥\leq {\left[\frac{\nu }{B(\nu )\Gamma (\nu +1)}+\frac{1-\nu }{B(\nu )}\right]}^{n}\varphi_{5}^{n}∥T_{n-1}-T∥ \end{array}$$ (61)

$$\begin{array}{c} ∥Z6_{n}∥\leq {\left[\frac{\nu }{B(\nu )\Gamma (\nu +1)}+\frac{1-\nu }{B(\nu )}\right]}^{n}\varphi_{6}^{n}∥R_{n-1}-R∥ \end{array}$$ (62)

As a result, we observe that *Zj*_*n*_(*t*) → 0 as *n* → ∞ for $j\in N_{1}^{6}$ and *φ* < 1 which complete the proof.

**Theorem 5.3** If the following is true, then the modified ABC model(8) has a unique solution:

$$\begin{array}{c} {}[\frac{\nu }{B(\nu )\Gamma (\nu +1)}+\frac{1-\nu }{B(\nu )}]\varphi_{j}\leq 1,j\in N_{1}^{6}. \end{array}$$

**Proof.** Assuming that the solution is dual in nature and non-unique, we can consider $\tilde{S}\left(t\right)$, $\tilde{E}\left(t\right)$, $\tilde{A}\left(t\right)$,$\tilde{C}\left(t\right)$,$\tilde{T}\left(t\right)$,$\tilde{R}\left(t\right)$, as alternative solutions. Then, we have:

$$\begin{array}{c} \begin{array}{cc} \tilde{S}\left(t\right) & =\tilde{S}\left(0\right)+\frac{1-\nu }{B(\nu )}\kappa_{1}(t,\tilde{S}(t))+\frac{\nu }{B(\nu )\Gamma (\nu )}\int_{0}^{t}{\left(t-\vartheta \right)}^{\nu -1}\kappa_{1}(\vartheta ,\tilde{S}(\vartheta ))d\vartheta \\ & -\frac{1-\nu }{B(\nu )}\kappa_{1}^{0}(t,\tilde{S}(t))(1+\frac{\gamma_{\nu }}{\Gamma (\nu +1)}t^{\nu }), \end{array} \end{array}$$ (63)

$$\begin{array}{c} \begin{array}{cc} \tilde{E}\left(t\right) & =\tilde{E}\left(0\right)+\frac{1-\nu }{B(\nu )}\kappa_{2}(t,\tilde{E}(t))+\frac{\nu }{B(\nu )\Gamma (\nu )}\int_{0}^{t}{\left(t-\vartheta \right)}^{\nu -1}\kappa_{2}(\vartheta ,\tilde{E}(\vartheta ))d\vartheta \\ & -\frac{1-\nu }{B(\nu )}\kappa_{2}^{0}(t,\tilde{E}(t))(1+\frac{\gamma_{\nu }}{\Gamma (\nu +1)}t^{\nu }), \end{array} \end{array}$$ (64)

$$\begin{array}{c} \begin{array}{cc} \tilde{A}\left(t\right) & =\tilde{A}\left(0\right)+\frac{1-\nu }{B(\nu )}\kappa_{3}(t,\tilde{A}(t))+\frac{\nu }{B(\nu )\Gamma (\nu )}\int_{0}^{t}{\left(t-\vartheta \right)}^{\nu -1}\kappa_{3}(\vartheta ,\tilde{A}(\vartheta ))d\vartheta \\ & -\frac{1-\nu }{B(\nu )}\kappa_{3}^{0}(t,\tilde{A}(t))(1+\frac{\gamma_{\nu }}{\Gamma (\nu +1)}t^{\nu }), \end{array} \end{array}$$ (65)

$$\begin{array}{c} \begin{array}{cc} \tilde{C}\left(t\right) & =\tilde{C}\left(0\right)+\frac{1-\nu }{B(\nu )}\kappa_{4}(t,\tilde{C}(t))+\frac{\nu }{B(\nu )\Gamma (\nu )}\int_{0}^{t}{\left(t-\vartheta \right)}^{\nu -1}\kappa_{4}(\vartheta ,\tilde{C}(\vartheta ))d\vartheta \\ & -\frac{1-\nu }{B(\nu )}\kappa_{4}^{0}(t,\tilde{C}(t))(1+\frac{\gamma_{\nu }}{\Gamma (\nu +1)}t^{\nu }), \end{array} \end{array}$$ (66)

$$\begin{array}{c} \begin{array}{cc} \tilde{T}\left(t\right) & =\tilde{T}\left(0\right)+\frac{1-\nu }{B(\nu )}\kappa_{5}(t,\tilde{T}(t))+\frac{\nu }{B(\nu )\Gamma (\nu )}\int_{0}^{t}{\left(t-\vartheta \right)}^{\nu -1}\kappa_{5}(\vartheta ,T(\vartheta ))d\vartheta \\ & -\frac{1-\nu }{B(\nu )}\kappa_{5}^{0}(t,\tilde{T}(t))(1+\frac{\gamma_{\nu }}{\Gamma (\nu +1)}t^{\nu }), \end{array} \end{array}$$ (67)

$$\begin{array}{c} \begin{array}{cc} \tilde{R}\left(t\right) & =\tilde{R}\left(0\right)+\frac{1-\nu }{B(\nu )}\kappa_{6}(t,\tilde{R}(t))+\frac{\nu }{B(\nu )\Gamma (\nu )}\int_{0}^{t}{\left(t-\vartheta \right)}^{\nu -1}\kappa_{6}(\vartheta ,\tilde{R}(\vartheta ))d\vartheta \\ & -\frac{1-\nu }{B(\nu )}\kappa_{6}^{0}(t,\tilde{R}(t))(1+\frac{\gamma_{\nu }}{\Gamma (\nu +1)}t^{\nu }), \end{array} \end{array}$$ (68)

Now, we write

$$\begin{array}{cc} ∥S-\tilde{S}∥ & =∥\frac{1-\nu }{B(\nu )}\kappa_{1}(t,S(t))+\frac{\nu }{B(\nu )\Gamma (\nu )}\int_{0}^{t}{\left(t-\vartheta \right)}^{\nu -1}\kappa_{1}(\vartheta ,S(\vartheta ))d\vartheta \\ \; & -\frac{1-\nu }{B(\nu )}\kappa_{1}^{0}(t,S(t))(1+\frac{\gamma_{\nu }}{\Gamma (\nu +1)}t^{\nu })\\ \; & -[\frac{1-\nu }{B(\nu )}\kappa_{1}(t,\tilde{S}(t))+\frac{\nu }{B(\nu )\Gamma (\nu )}\int_{0}^{t}{\left(t-\vartheta \right)}^{\nu -1}\kappa_{1}(\vartheta ,\tilde{S}(\vartheta ))d\vartheta \\ \; & -\frac{1-\nu }{B(\nu )}\kappa_{1}^{0}(t,\tilde{S}(t))(1+\frac{\gamma_{\nu }}{\Gamma (\nu +1)}t^{\nu })]\\ \; & =\frac{\nu }{B(\nu )\Gamma (\nu )}\int_{0}^{t}{\left(t-\vartheta \right)}^{\nu -1}∥\kappa_{1}(\vartheta ,S(\vartheta ))-\kappa_{1}(\vartheta ,\tilde{S}(\vartheta ))∥d\vartheta \\ \; & +\frac{1-\nu }{B(\nu )}∥\kappa_{1}(t,S(t))-\kappa_{1}(t,\tilde{S}(t))∥\\ \; & \leq \frac{\nu }{B(\nu )\Gamma (\nu )}\int_{0}^{t}{\left(t-\vartheta \right)}^{\nu -1}\varphi_{1}∥S-\tilde{S}∥d\vartheta \\ \; & +\frac{1-\nu }{B(\nu )}\varphi_{1}∥S-\tilde{S}∥\\ & \leq [\frac{\nu }{B(\nu )\Gamma (\nu +1)}+\frac{1-\nu }{B(\nu )}]\varphi_{1}∥S-\tilde{S}∥. \end{array}$$ (69)

and so

$$\begin{array}{c} {}[1-(\frac{\nu }{B(\nu )\Gamma (\nu +1)}+\frac{1-\nu }{B(\nu )})\varphi_{1}]∥S-\tilde{S}∥\leq 0, \end{array}$$ (70)

when $∥S-\tilde{S}∥=0,$ the inequality (70) holds, suggesting that S is equal to $\tilde{S}.$ Similarly, we have

$$\begin{array}{c} {}[1-(\frac{\nu }{B(\nu )\Gamma (\nu +1)}+\frac{1-\nu }{B(\nu )})\varphi_{1}]∥E-\tilde{E}∥\leq 0, \end{array}$$ (71)

$$\begin{array}{c} {}[1-(\frac{\nu }{B(\nu )\Gamma (\nu +1)}+\frac{1-\nu }{B(\nu )})\varphi_{1}]∥A-\tilde{A}∥\leq 0, \end{array}$$ (72)

$$\begin{array}{c} {}[1-(\frac{\nu }{B(\nu )\Gamma (\nu +1)}+\frac{1-\nu }{B(\nu )})\varphi_{1}]∥C-\tilde{C}∥\leq 0, \end{array}$$ (73)

$$\begin{array}{c} {}[1-(\frac{\nu }{B(\nu )\Gamma (\nu +1)}+\frac{1-\nu }{B(\nu )})\varphi_{1}]∥T-\tilde{T}∥\leq 0, \end{array}$$ (74)

and

$$\begin{array}{c} {}[1-(\frac{\nu }{B(\nu )\Gamma (\nu +1)}+\frac{1-\nu }{B(\nu )})\varphi_{1}]∥R-\tilde{R}∥\leq 0, \end{array}$$ (75)

As a result, the HBV model (8) solution demonstrates the uniqueness property.

## 6 Numerical scheme

Examining the first equation of (8), we have

$$\begin{array}{c} \begin{array}{cc} S & =S_{0}+\frac{1-\nu }{B(\nu )}\kappa_{1}(t,S(t))+\frac{\nu }{B(\nu )\Gamma (\nu )}\int_{0}^{t}{\left(t-\vartheta \right)}^{\nu -1}\kappa_{1}(\vartheta ,S(\vartheta ))d\vartheta \\ & -\frac{1-\nu }{B(\nu )}\kappa_{1}(0,S(0))(1+\frac{\gamma_{\nu }}{\Gamma (\nu +1)}t^{\nu }). \end{array} \end{array}$$ (76)

We are using Lagrange’s interpolation polynomials to generate a numerical method for (76). Replacing t by *t*_*n*+1_, we get

$$\begin{array}{c} \begin{array}{cc} S & =S_{0}+\frac{1-\nu }{B(\nu )}\kappa_{1}(t_{n},S(t_{n}))+\frac{\nu }{B(\nu )\Gamma (\nu )}\int_{0}^{t_{n+1}}{\left(t_{n+1}-\vartheta \right)}^{\nu -1}\kappa_{1}(\vartheta ,S(\vartheta ))d\vartheta \\ & -\frac{1-\nu }{B(\nu )}\kappa_{i}^{*}(0,S(0))(1+\frac{\gamma_{\nu }}{\Gamma (\nu +1)}{t_{n}}^{\nu }). \end{array} \end{array}$$ (77)

By the Lagrange’s interpolation, we have

$$\begin{array}{c} \begin{array}{cc} \kappa_{1}(t,S(t)) & =\frac{\kappa_{1}(t_{b},S(t_{b}))(t-t_{b-1})}{t_{b}-t_{b-1}}-\frac{\kappa_{1}(t_{b-1},S(t_{b-1}))(t-t_{b})}{t_{b}-t_{b-1}}\\ & =\frac{\kappa_{1}(t_{b},S(t_{b}))(t-t_{b-1})}{h}-\frac{\kappa_{1}(t_{b-1},S(t_{b-1}))(t-t_{b})}{h}. \end{array} \end{array}$$ (78)

By the help of (77) and (78), we have

$$\begin{array}{c} \begin{array}{cc} S(t_{b+1}) & =S_{0}+\frac{1-\nu }{B(\nu )}\kappa_{1}(t_{b},S(t_{b}))\\ & +\frac{\nu }{B(\nu )\Gamma (\nu )}\sum_{\iota =1}^{n}[\frac{\kappa_{1}(t_{\iota },u(t_{\iota }))}{h}\int_{t_{b}}^{t_{b+1}}(\vartheta -t_{\iota -1}){\left(t_{n+1}-\vartheta \right)}^{\nu -1}d\vartheta \\ & -\frac{\kappa_{1}(t_{\iota -1},\varpi_{\iota }(t_{\iota -1}))}{h}\int_{t_{b}}^{t_{n+1}}(\vartheta -t_{\iota }){\left(t_{n+1}-\vartheta \right)}^{\nu -1}d\vartheta ]\\ & -\frac{1-\nu }{B(\nu )}\kappa_{1}(0,S(0))(1+\frac{\gamma_{\nu }}{\Gamma (\nu +1)}{t_{b}}^{\nu }). \end{array} \end{array}$$ (79)

Solving the integrals, we get

$$\begin{array}{c} \begin{array}{cc} S_{b+1} & =S_{0}+\frac{1-\nu }{B(\nu )}\kappa_{1}(t_{b},S(t_{b}))\\ & +\frac{\nu h^{\nu }}{\Gamma (\nu +2)}\sum_{†=1}^{b}[\kappa_{1}(t_{\iota },S(t_{\iota }))({\left({ℑ}_{1}\right)}^{\nu }({ℑ}_{2})\\ & -{\left({ℑ}_{3}\right)}^{\nu }({ℑ}_{4}))-\kappa_{1}(t_{\iota -1},S_{\iota -1})({\left({ℑ}_{3}+1\right)}^{\nu +1}\\ & -({ℑ}_{1}+\nu ){\left({ℑ}_{3}\right)}^{\nu })]-\frac{1-\nu }{B(\nu )}\kappa_{1}(0,S(0))(1+\frac{\gamma_{\nu }}{\Gamma (\nu +1)}{\left(bh\right)}^{\nu }). \end{array} \end{array}$$ (80)

Similarly

$$\begin{array}{c} \begin{array}{cc} E_{b+1} & =E_{0}+\frac{1-\nu }{B(\nu )}\kappa_{2}(t_{b},E(t_{b}))\\ & +\frac{\nu h^{\nu }}{\Gamma (\nu +2)}\sum_{†=1}^{b}[\kappa_{2}(t_{\iota },E(t_{\iota }))({\left({ℑ}_{1}\right)}^{\nu }({ℑ}_{2})\\ & -{\left({ℑ}_{3}\right)}^{\nu }({ℑ}_{4}))-\kappa_{2}(t_{\iota -1},E_{\iota -1})({\left({ℑ}_{3}+1\right)}^{\nu +1}\\ & -({ℑ}_{1}+\nu ){\left({ℑ}_{3}\right)}^{\nu })]-\frac{1-\nu }{B(\nu )}\kappa_{2}(0,E(0))(1+\frac{\gamma_{\nu }}{\Gamma (\nu +1)}{\left(bh\right)}^{\nu }). \end{array} \end{array}$$ (81)

$$\begin{array}{c} \begin{array}{cc} A_{b+1} & =A_{0}+\frac{1-\nu }{B(\nu )}\kappa_{3}(t_{b},A(t_{b}))\\ & +\frac{\nu h^{\nu }}{\Gamma (\nu +2)}\sum_{†=1}^{b}[\kappa_{3}(t_{\iota },A(t_{\iota }))({\left({ℑ}_{1}\right)}^{\nu }({ℑ}_{2})\\ & -{\left({ℑ}_{3}\right)}^{\nu }({ℑ}_{4}))-\kappa_{3}(t_{\iota -1},A_{\iota -1})({\left({ℑ}_{3}+1\right)}^{\nu +1}\\ & -({ℑ}_{1}+\nu ){\left({ℑ}_{3}\right)}^{\nu })]-\frac{1-\nu }{B(\nu )}\kappa_{3}(0,A(0))(1+\frac{\gamma_{\nu }}{\Gamma (\nu +1)}{\left(bh\right)}^{\nu }). \end{array} \end{array}$$ (82)

$$\begin{array}{c} \begin{array}{cc} C_{b+1} & =C_{0}+\frac{1-\nu }{B(\nu )}\kappa_{4}(t_{b},C(t_{b}))\\ & +\frac{\nu h^{\nu }}{\Gamma (\nu +2)}\sum_{†=1}^{b}[\kappa_{4}(t_{\iota },C(t_{\iota }))({\left({ℑ}_{1}\right)}^{\nu }({ℑ}_{2})\\ & -{\left({ℑ}_{3}\right)}^{\nu }({ℑ}_{4}))-\kappa_{4}(t_{\iota -1},C_{\iota -1})({\left({ℑ}_{3}+1\right)}^{\nu +1}\\ & -({ℑ}_{1}+\nu ){\left({ℑ}_{3}\right)}^{\nu })]-\frac{1-\nu }{B(\nu )}\kappa_{4}(0,C(0))(1+\frac{\gamma_{\nu }}{\Gamma (\nu +1)}{\left(bh\right)}^{\nu }). \end{array} \end{array}$$ (83)

$$\begin{array}{c} \begin{array}{cc} T_{b+1} & =T_{0}+\frac{1-\nu }{B(\nu )}\kappa_{5}(t_{b},T(t_{b}))\\ & +\frac{\nu h^{\nu }}{\Gamma (\nu +2)}\sum_{†=1}^{b}[\kappa_{5}(t_{\iota },T(t_{\iota }))({\left({ℑ}_{1}\right)}^{\nu }({ℑ}_{2})\\ & -{\left({ℑ}_{3}\right)}^{\nu }({ℑ}_{4}))-\kappa_{5}(t_{\iota -1},T_{\iota -1})({\left({ℑ}_{3}+1\right)}^{\nu +1}\\ & -({ℑ}_{1}+\nu ){\left({ℑ}_{3}\right)}^{\nu })]-\frac{1-\nu }{B(\nu )}\kappa_{5}(0,T(0))(1+\frac{\gamma_{\nu }}{\Gamma (\nu +1)}{\left(bh\right)}^{\nu }). \end{array} \end{array}$$ (84)

$$\begin{array}{c} \begin{array}{cc} R_{b+1} & =R_{0}+\frac{1-\nu }{B(\nu )}\kappa_{6}(t_{b},R(t_{b}))\\ & +\frac{\nu h^{\nu }}{\Gamma (\nu +2)}\sum_{†=1}^{b}[\kappa_{6}(t_{\iota },R(t_{\iota }))({\left({ℑ}_{1}\right)}^{\nu }({ℑ}_{2})\\ & -{\left({ℑ}_{3}\right)}^{\nu }({ℑ}_{4}))-\kappa_{6}(t_{\iota -1},R_{\iota -1})({\left({ℑ}_{3}+1\right)}^{\nu +1}\\ & -({ℑ}_{1}+\nu ){\left({ℑ}_{3}\right)}^{\nu })]-\frac{1-\nu }{B(\nu )}\kappa_{6}(0,R(0))(1+\frac{\gamma_{\nu }}{\Gamma (\nu +1)}{\left(bh\right)}^{\nu }), \end{array} \end{array}$$ (85)

where ℑ_1_ = *b* − † + 1, ℑ_2_ = *b* + 2 − † + *ν*, ℑ_3_ = *b* − †, ℑ_4_ = *b* + 2 − † + 2*ν*.

## 7 Numerical results and discussion

**Fig 7:**
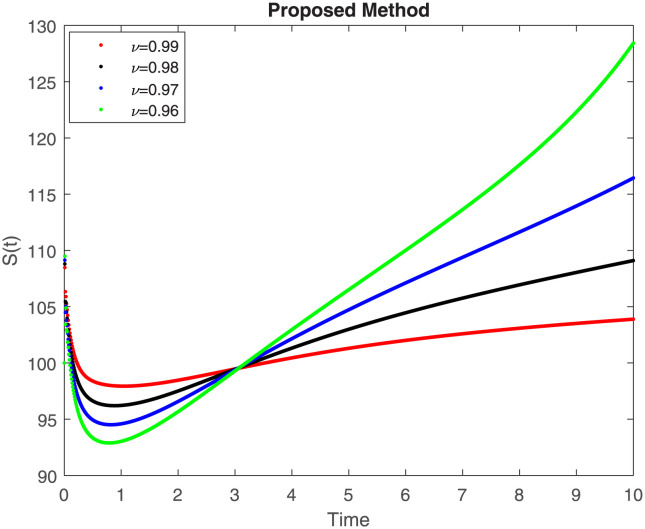
Graphical representation of S(t) class when *R*_0_ < 1 under MABC.

**Fig 8:**
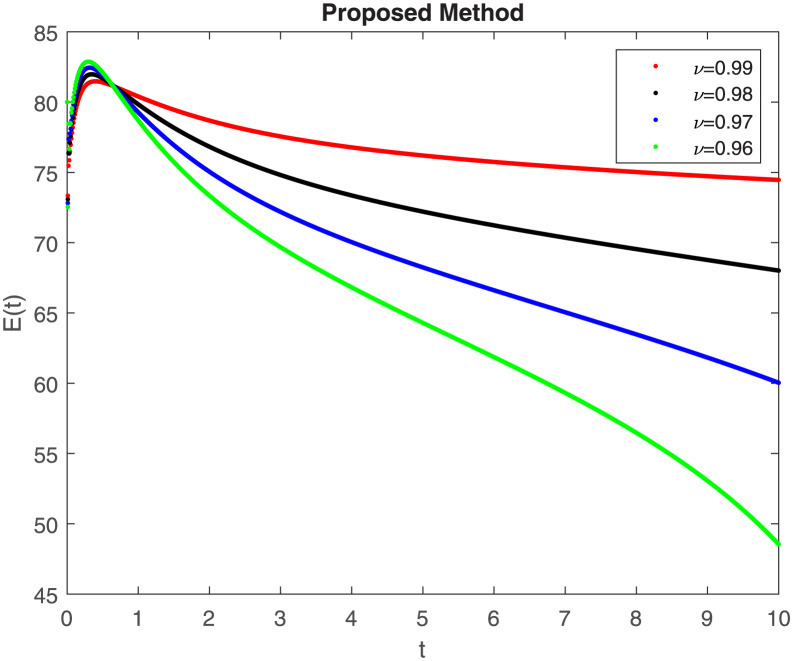
Graphical representation of E(t) class when *R*_0_ < 1 under MABC.

**Fig 9:**
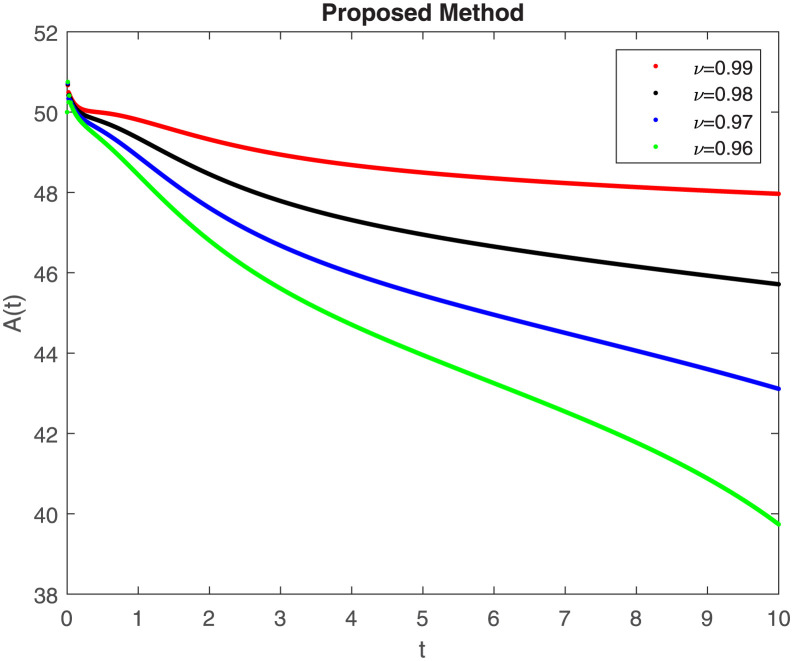
Graphical representation of A(t) class when *R*_0_ < 1 under MABC.

**Fig 10:**
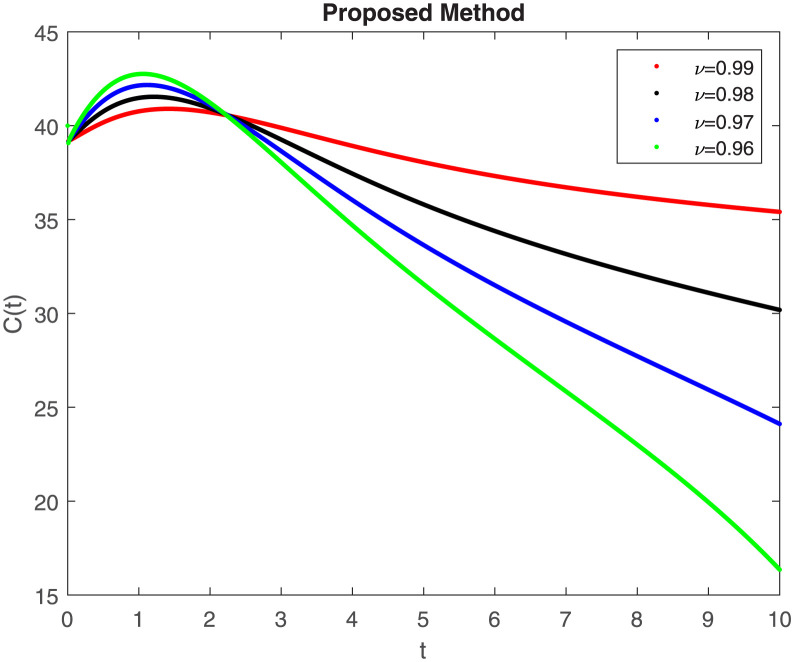
Graphical representation of C(t) when *R*_0_ < 1 under MABC.

**Fig 11:**
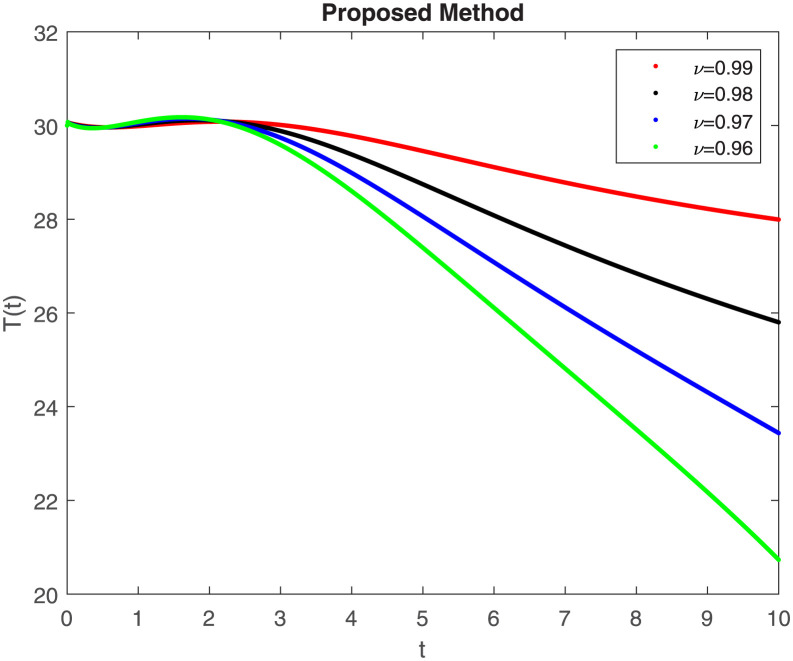
Graphical representation of T(t) when *R*_0_ < 1 under MABC.

**Fig 12:**
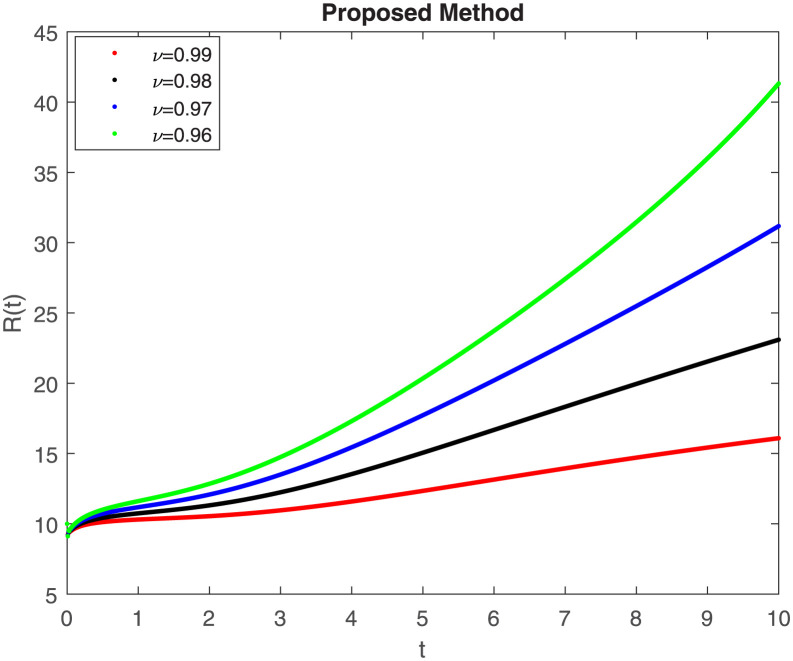
Graphical representation of R(t) class when *R*_0_ < 1 under MABC.

**Fig 13:**
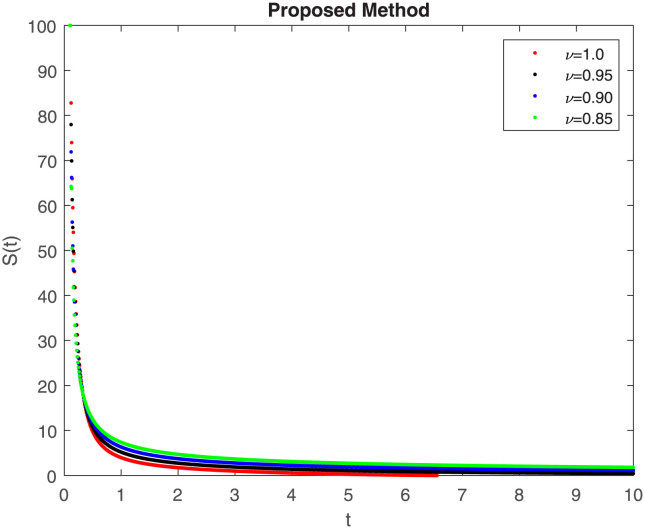
Graphical representation of S(t) class when *R*_0_ < 1 under ABC.

**Fig 14:**
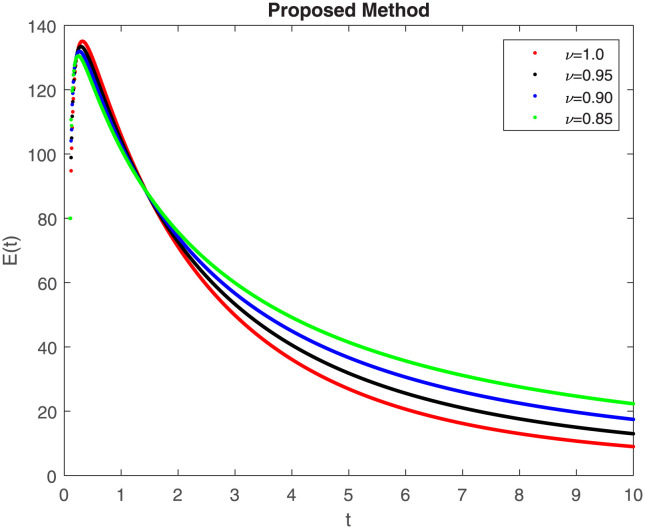
Graphical representation of E(t) class when *R*_0_ < 1 under ABC.

**Fig 15:**
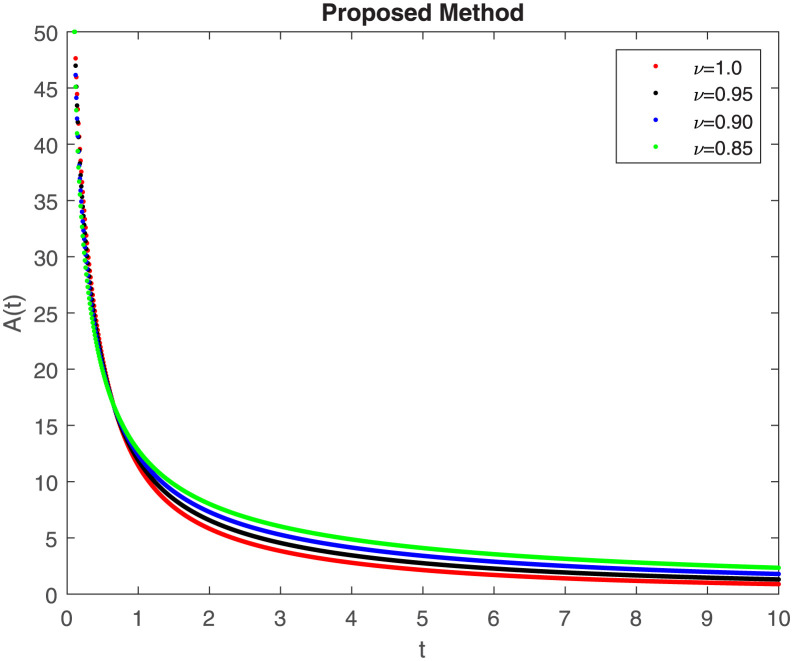
Graphical representation of A(t) class when *R*_0_ < 1 under ABC.

**Fig 16:**
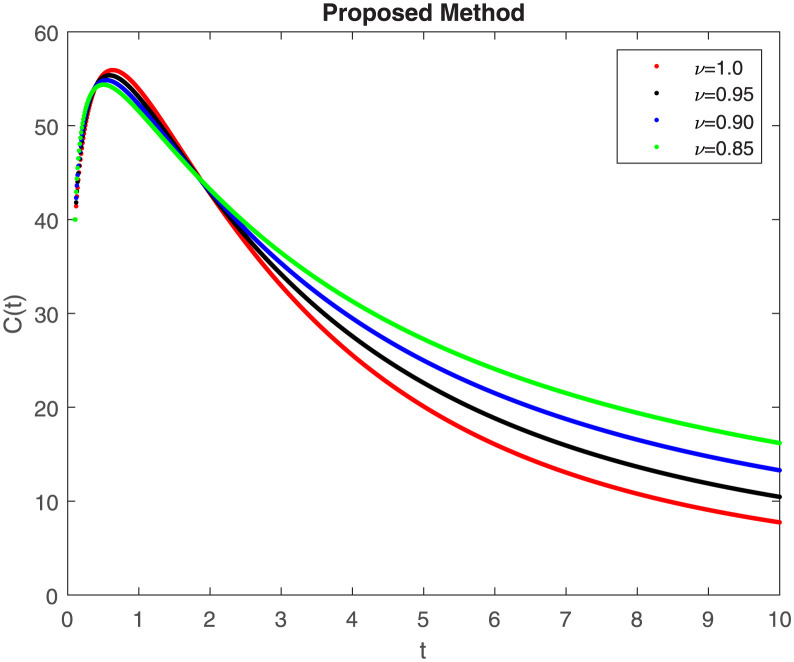
Graphical representation of C(t) class when *R*_0_ < 1 under ABC.

**Fig 17:**
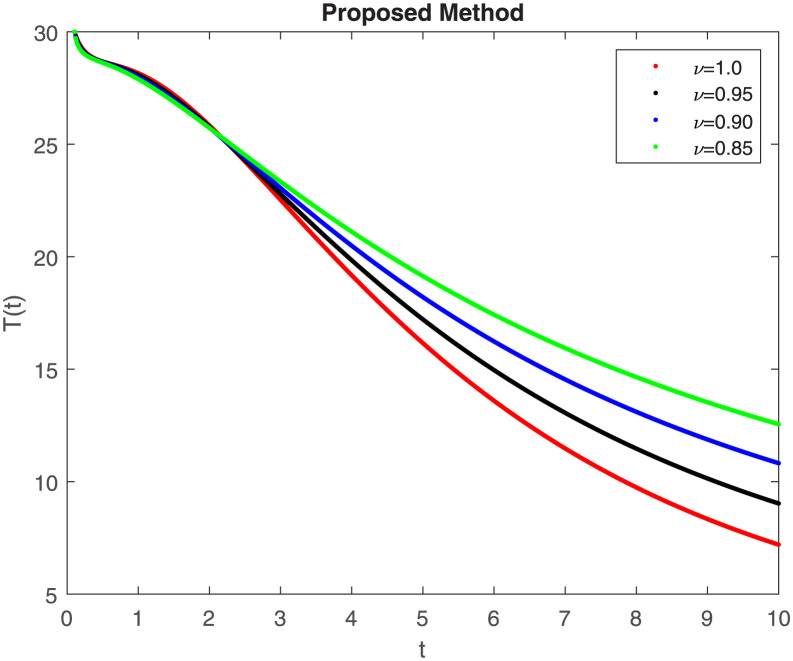
Graphical representation of T(t) class when *R*_0_ < 1 under ABC.

**Fig 18:**
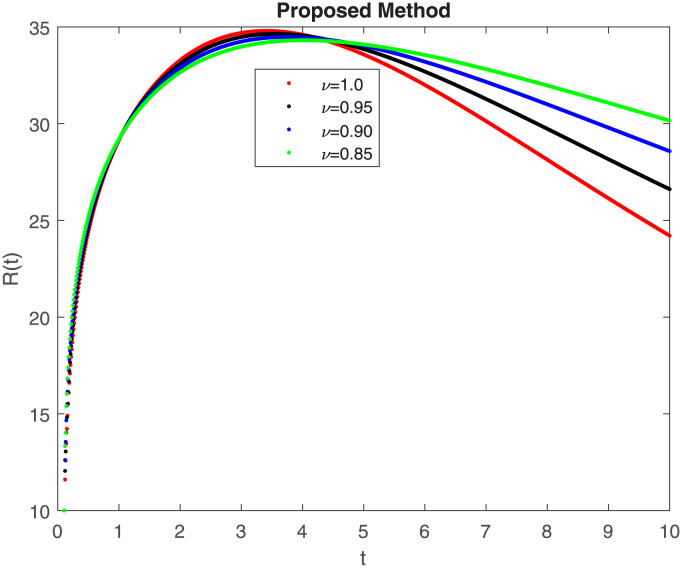
Graphical representation of R(t) class when *R*_0_ < 1 under ABC.

The fractional-order epidemiological model (8) for the hepatitis B virus is studied in this section using numerical simulations to examine its temporal dynamical behavior. Evaluating the analytical work’s validity through large-scale numerical simulation and proving the work’s viability is essential. Atangana-Baleanu in the Caputo sense combined with Mittag-Leffler law is used to calculate the numerical outcomes of the model for various fractional values based on the steady-state point. These simulations show that the dynamics of the model are impacted by a change in value. Additionally, the fractional value findings show greater efficiency when contrasted with the standard derivative. It makes it possible to estimate the ideal value for disease control with greater accuracy. The real data parameters are [31]: *c* = 0.33, *ω* = 0.32, *γ* = 0.5, *θ* = 0.52, *μ* = 0.27, *p*_1_ = 0.11, *c*_1_ = 0.082, *c*_2_ = 0.13, *σ* = 0.28, *δ*_1_ = 0.475, *δ*_2_ = 0.4, *ϕ* = 0.32, *d* = 0.175, *τ* = 0.483, *u*_2_ = 0.06. The dynamics of the HBV model employing the non-singular fractional derivative of the Modified Atangana-Baleanu type are shown in Figs (7)–(12) for various values of *ν* ∈ (0, 1). The Figures demonstrate, HBV model (8) using the MABC technique output the crossover phenomenon in all compartments of the model, which show the dynamic change in progression of HBV transmission. The figures illustrate how different compartments go through peaks and decline, reflecting interaction between illness, therapy and immunity in spreading and controlling HBV. The different dynamics present when using different *ν* values indicate the sensitivity of the model to the parameters and highlight the significance of considering these factors in HBV transmission dynamics understanding. The crossover behavior in the system results in altering the trend for the population dynamics in time with these changes occurring depending on the value of the fractional-order derivative *ν*. With the use of the non-singular fractional derivative of the Atangana-Baleanu type, Figs(13)–(18) illustrate the dynamics of the HBV model for various values of *ν* ∈ (0, 1). For different fractional orders, Figs (7) and (13) shows the dynamics for S(t). The class population is gradually growing in proportion to the derivative’s decreasing order. For various fractional orders *ν*, the dynamics for the E(t),A(t),C(t), and T(t) respectively, are expressed in Figs (8)–(11). The population of the classes gradually declines in proportion to the derivative’s decreasing order. Similarly, we see a progressive increase in the class population to the derivative orders reduction in Fig (12), which shows the dynamics for R(t) for the fractional orders *ν*. A graphic comparison between our outcomes and the ABC operator has been made. The MABC fractional derivative gives us access to additional information and solutions related to the proposed model. The figures illustrate the role of vaccination in protecting the exposed population by reducing chronic infections, preventing new infections, and managing/treating HBV cases. The patterns observed in the susceptible, exposed, acute infected, chronic infected, treated, and recovered compartment with different values of *ν* suggest the effectiveness of vaccination in controlling HBV transmission dynamics. As memory effects are shown in biological systems, we have seen that fractional order modeling of those systems can be advantageous. *ν* is an important parameter in the model dynamics even though it is not yet a biological parameter (8). By investigating the dynamics of these compartments in the HBV model with the MABC method, researchers will obtain information about the development of HBV infection, the applicability of interventions, and the overall effect on disease transmission dynamics.

## 6 Conclusion

In this work, we suggested a SEACTR model of viral infection with hepatitis B, including two controls: immunization and treatment. We employ the HBV model with the fractional operator to verify the system’s dynamic behavior for constant controls. In our proposed problem, we considered the recently developed modified ABC operator. This new operator has extra benefits over the prior ABC operator, including initialization and well-posedness. We think this operator has given researchers a new direction to pursue in their work. The existence of solutions was ascertained by applying Leray Shauder’s approach. The Lyapunov function is used to examine the fractional order model globally stable. Using Lagrange’s interpolation polynomial, a novel numerical technique was created and then applied to a mathematical model of HBV. We noted that the results are more grounded in reality and that the scheme has additional utility for studying dynamical systems. The impact of fractional order is demonstrated using a numerical simulation that examines the vaccination’s impacts on the community. Additionally, the study demonstrates that vaccination and treatment effects are more notable for the entire population. The MABC Hepatitis B model exhibits good accuracy when it comes to stability, with comparatively smaller fractional orders. Reduced fractional order improved HBV control in all of the numerical simulations. The aforementioned model will be investigated in the future utilizing optimum control analysis and stochastic analysis.
